# Pet keeping in childhood and asthma and allergy among children in Tianjin area, China

**DOI:** 10.1371/journal.pone.0197274

**Published:** 2018-05-16

**Authors:** Shugang Luo, Yuexia Sun, Jing Hou, Xiangrui Kong, Pan Wang, Qingnan Zhang, Jan Sundell

**Affiliations:** Tianjin Key Lab. of Indoor Air Environmental Quality Control, School of Environmental Science and Engineering, Tianjin University, Tianjin, China; Ospedale S. Corona, ITALY

## Abstract

This study aims to find out the relationship between pet keeping in childhood and asthma and allergy among children aged 0–8 years old in Tianjin, China. Parental or guardians reports of 7360 children were analyzed. 1490 (21.6%) families kept pets at the time of the survey (current), among them 4.0% cats, and 14.7% dogs. For the first year of life of children (early), 1196 (18.4%) families kept pets, and among them 3.2% cats, and 13.7% dogs. Exposure to a pet in early childhood significantly increased the risk of current wheeze, current dry cough, and diagnosed rhinitis. 17.9% of parents reported an avoidance behavior, i.e., had removed or refrained a pet due to asthma or allergy in the family. After adjustment for avoidance behavior, the negative effect of pet keeping on children’s health became even more obvious, with e.g. an AOR of 3.37 (1.58–7.19) for diagnosed asthma, 3.60 (2.07–6.27) for diagnosed rhinitis, 1.92 (1.31–2.81) for diagnosed eczema. A dose-response relationship between pet keeping at current and current wheeze, current eczema and diagnosed eczema was found. In conclusion, pet exposure in early life of children in Tianjin is a risk factor for asthma and allergies among children aged 0–8 years old.

## Introduction

The impact of pet keeping on the development of allergic diseases is discussed [1]. Some studies support the view that pet keeping in early life decreases the risk of asthma and allergies in children. For example, Hesselmar at al [2] found that exposure to pets during the first year of life decreased the risk of allergic rhinitis at 7–9 years and of asthma at 12–13 years. Ownby et al [3] even found that children exposed to two or more dogs or cats had the lowest prevalence of any skin prick test compared with children exposed to less pets. On the other hand, some studies found that pet keeping in early life of children is significantly positively associated with symptoms in later life. For example, Wahn et al [4] found that children who exposure to higher cat allergen (150 ng/gm vs 64 ng/gm) were more likely to get allergy. Lombardi et al [5] found exposure to cat in the first year of life significantly increased the risk of current wheeze. A Swedish study found that the protective effects of pet exposure were due to avoidance behavior [6], i.e., allergic families intend to remove or refrain from pets.

Most of early studies are based on Western populations or populations of industrialized regions. Some studies also have been conducted in Asia. In 1999, Zheng et al [7] conducted a case-control study in Beijing, China and found that dog or cat keeping was an important risk factor for children’s asthma. In 2001, Salo et al [8] conducted a study in Wuhan, China, and revealed that keeping pets at current was positively associated with persistent cough and wheeze; exposure to pets in early life significantly increased the risk of diagnosed asthma. In order to study associations between the home environment and children’s asthma and allergies, a national study “China, Children, Health and Home (CCHH)” has been conducted since 2010. This paper aims to find out the relationship between early exposure to pets and allergic symptoms among children in Tianjin, China.

## Methods

Since April 2013, randomly selected kindergartens, daycare centers and primary schools were invited to participate in this study. With the help from the teachers, questionnaires were distributed to parents of children aged 0–8 years old.

The questionnaire was developed from the Dampness in Buildings and Health (DBH) study in Sweden [9], which has been used in many countries and cities respectively [10]. However, questions on building characteristics were modified to better reflect the Chinese housing styles. The questions on children’s asthma and allergy are identical to core questions used in ISAAC (International Study on Asthma and Allergy of Child) study [11]. The good validity and reliability of the questionnaire were tested and reported in previous Chinese studies [12–13]. The entire questionnaire is shown in S1 and S2 Files. It consists of questions on children’s background information, life styles, home environment and health. Children’s health outcomes are wheeze in the last 12 months (current wheeze), dry cough in the last 12 months (current dry cough), diagnosed asthma, rhinitis in the last 12 months (current rhinitis), diagnosed rhinitis, eczema in the last 12 months (current eczema), and diagnosed eczema. With regard to pet keeping, parents were asked whether their family kept pets in the current home and/or in the first year of life of children; and if so, what type of pets: dog, cat, rodent (rabbit, hamster), bird, fish?

In this study, the database was divided into four groups of different pet keeping behavior (pet keeping all the time, never, at birth but not current, not at birth but current) to assess the avoidance behavior as Bornehag et al did in Sweden [6]. Moreover, parents were also asked to describe their avoidance behaviors: whether they removed or refrained to have a pet due to asthma or allergy among their families.

### Statistics

Logistic regression models were used to evaluate the association of pet exposure with asthma and allergies among children. In the multivariate analyses we adjusted for gender, age, total household income, family allergic history, location, dampness and avoidance behavior. A p-value of less than 0.05 was accepted to be of significance. All statistical analyses were performed with the SPSS (version 22).

### Ethical statement

Ethical approval was obtained from the Research Office at Tianjin University. Our participants provided verbal informed consent to participate in this study. This study is anonymous questionnaire survey. It involves no risk to the subjects. The study could not practicably be carried out with written consent. Completed surveys were used to reflect participant consents. These surveys were stored in a lock cabinet. Research Office at Tianjin University approved this consent procedure.

## Results

7865 parents answered the questionnaire survey, with a response rate of 78%. Ages were not reported for 204 children, and 295 children were outside 0 to 8 age boundary. Therefore, our final analysis was of 7366 children, among which 3539 resided in inner city, 1483 in suburban, 1755 in rural areas. Demographic information, health outcomes and exposure to pets are summarized in Table 1. The highest prevalence of asthma and allergies was reported by people living in city, followed by suburban and rural areas. Pet keeping had an opposite trend. The highest rate of pet keeping was reported from rural areas. However, people living in city had fish as pets more often. Dog is the most popular pet.

**Table 1:** Demographic information, health outcomes and exposure to pets of the investigated population, n (%).

			Home location			
			Total	Rural	Suburban	Urban
Demographic information						
	Gender					
		Male	3780 (51.9)	916 (52.9)	756 (51.5)	1801 (51.5)
		Female	3499 (48.1)	815 (47.1)	712 (48.5)	1699 (48.5)
	Age					
		0–2 years old	225 (3.1)	65 (3.7)	44 (3.0)	102 (2.9)
		3–5 years old	3238 (44.0)	751 (42.8)	677 (45.7)	1549 (43.8)
		6–8 years old	3903 (53.0)	939 (53.5)	762 (51.4)	1888 (53.3)
	Members of family have asthma or allergies		948 (14.1)	113 (7.0)	164 (11.7)	625 (18.4)
	Dampness in home		1370 (23.0)	363 (26.8)	318 (25.6)	637 (20.7)
	Total income (RMB)					
		<30 thousand	1123 (17.4)	482 (31.8)	249 (18.7)	288 (8.9)
		30–50 thousand	1516 (23.5)	619 (40.8)	370 (27.7)	434 (13.5)
		50–100 thousand	1863 (28.9)	342 (22.5)	429 (32.1)	989 (30.7)
		100–200 thousand	1381 (21.4)	58 (3.8)	201 (15.1)	1071 (33.2)
		>200 thousand	572 (8.9)	16 (1.1)	86 (6.4)	444 (13.8)
Health outcomes						
	Current wheeze		333 (4.9)	41 (2.5)	56 (4.1)	199 (5.9)
	Current dry cough		937 (13.6)	148 (8.9)	171 (12.0)	556 (16.2)
	Diagnosed asthma		302 (4.4)	40 (2.4)	44 (3.1)	193 (5.7)
	Current rhinitis		2002 (29.8)	324 (20.3)	363 (26.4)	1221 (36)
	Diagnosed rhinitis		636 (9.5)	72 (4.4)	110 (7.9)	416 (12.4)
	Current eczema		998 (14.9)	198 (12.4)	189 (13.7)	558 (16.5)
	Diagnosed eczema		2624 (39.1)	460 (29)	501 (36.2)	1552 (45.8)
Pets exposure						
	Keeping pets at time of survey (current)		1490 (21.6)	678 (40.8)	279 (19.5)	458 (13.3)
		Cat	224 (4.0)	135 (12.1)	31 (2.6)	49 (1.6)
		Dog	929 (14.7)	526 (34.9)	180 (13.5)	178 (5.6)
		Rodent	111 (2.0)	46 (4.5)	12 (1.0)	50 (1.6)
		Bird	141 (2.5)	50 (4.8)	33 (2.8)	54 (1.8)
		Fish	341 (5.9)	39 (3.7)	76 (6.2)	207 (6.5)
	Keeping pets in the first year of life (early)		1196 (18.4)	509 (32.8)	257 (19.0)	377 (11.5)
		Cat	177 (3.2)	85 (7.5)	33 (2.9)	49 (1.7)
		Dog	845 (13.7)	421 (28.8)	187 (14.6)	206 (6.6)
		Rodent	53 (1.0)	16 (1.5)	11 (1.0)	22 (0.8)
		Bird	83 (1.5)	25 (2.3)	18 (1.6)	37 (1.3)
		Fish	175 (3.2)	18 (1.7)	39 (3.4)	106 (3.5)

In this study, children who had pets at birth but not currently showed the highest prevalence of diagnosed asthma, current rhinitis, current eczema and diagnosed eczema, while children who had pets currently but not at birth showed the highest prevalence of current wheeze and current dry cough. Children who had pets all the time showed the highest prevalence rate of diagnosed rhinitis (Table 2). S1 Table shows the prevalence of allergic symptoms for different “avoidance behavior”. The highest prevalence of allergic symptoms appeared in the group with an avoidance behavior (followed by the group that had pets at home currently, and the group neither having pets nor having avoidance behavior). And it also revealed that the influence of behavior is more clear in urban areas.

**Table 2:** Prevalence (n, %) of asthma and allergy among children with different pets keeping status.

	Prevalence, n (%)			
	Group Ⅰ^a^, n = 4707	Group Ⅱ^b^, n = 342	Group Ⅲ^c^, n = 740	Group Ⅳ^d^, n = 598
Current wheeze	212 (4.7)	**22 (6.9)**	25 (3.6)	32 (5.8)
Current dry cough	611 (13.4)	**64 (19.5)**	86 (12.0)	67 (11.8)
Diagnosed asthma	209 (4.6)	13 (3.9)	**33 (4.7)**	21 (3.7)
Current rhinitis	1362 (30.2)	92 (29.1)	**210 (30.4)**	144 (26.1)
Diagnosed rhinitis	437 (9.8)	25 (7.7)	59 (8.3)	**57 (10.3)**
Current eczema	642 (14.3)	47 (14.6)	**128 (18.4)**	96 (17.4)
Diagnosed eczema	1811 (40.4)	117 (35.8)	**296 (42.0)**	214 (39.2)

^a^Group Ⅰ: Never have pets.

^b^Group Ⅱ: Having pets at current, but not early.

^c^Group Ⅲ: Having pets at early, but not currently.

^d^Group Ⅳ: Having pets all the time.

Table 3 shows there is no “protective” effect of neither pet keeping currently nor pet keeping early for asthma and allergies. It indicated that pet keeping especially cats exposure was a significant risk factor for diagnosed asthma and diagnosed eczema. The effect of keeping pets became even more negative after adjusting for avoidance behavior (Table 4).

**Table 3:** Adjusted odds ratios of pet keeping for asthma and allergies among children^a^.

	Current wheeze	Current dry cough	Diagnosed asthma	Current rhinitis	Diagnosed rhinitis	Current eczema	Diagnosed eczema
No pets keeping (reference)	1	1	1	1	1	1	1
Pet keeping currently	1.28(0.81–2.04)	1.04(0.76–1.43)	1.13(0.69–1.83)	0.99(0.77–1.28)	1.21(0.85–1.72)	1.25(0.92–1.70)	1.24(0.98–1.58)
Cat	1.47(0.62–3.50)	1.01(0.58–1.74)	**3.12(1.58–6.16)**	1.12(0.74–1.70)	1.49(0.79–2.84)	**1.76(1.09–2.85)**	1.42(0.97–2.07)
Dog	1.37(0.85–2.22)	1.02(0.76–1.38)	1.28(0.79–2.06)	1.07(0.85–1.34)	1.27(0.89–1.81)	**1.33(1.00–1.76)**	1.22(0.99–1.50)
Rodent	2.22(0.98–5.07)	1.34(0.74–2.45)	**2.74(1.29–5.82)**	1.08(0.65–1.79)	1.56(0.78–3.14)	1.55(0.87–2.77)	1.42(0.88–2.28)
Bird	1.20(0.43–3.40)	0.97(0.50–1.85)	1.10(0.39–3.12)	1.11(0.68–1.80)	1.58(0.80–3.12)	1.33(0.73–2.42)	1.30(0.83–2.05)
Fish	1.35(0.78–2.32)	0.78(0.52–1.18)	0.71(0.36–1.43)	1.02(0.76–1.38)	1.43(0.96–2.14)	**1.66(1.18–2.32)**	**1.37(1.03–1.81)**
Pet keeping early	**1.70(1.07–2.71)**	1.32(0.96–1.84)	0.85(0.48–1.53)	0.91(0.69–1.20)	1.06(0.71–1.57)	0.96(0.68–1.36)	1.00(0.77–1.30)
Cat	**2.57(1.18–5.62)**	1.39(0.81–2.40)	**2.86(1.41–5.82)**	1.27(0.81–1.99)	1.87(0.99–3.54)	1.60(0.94–2.73)	1.16(0.76–1.77)
Dog	**1.59(1.00–2.51)**	1.12(0.84–1.50)	0.82(0.48–1.42)	0.95(0.76–1.20)	1.17(0.82–1.68)	1.29(0.98–1.70)	1.06(0.86–1.30)
Rodent	2.01(0.59–6.93)	**2.29(1.04–5.06)**	**3.80(1.37–10.53)**	0.51(0.21–1.20)	1.28(0.42–3.87)	1.53(0.64–3.65)	0.73(0.35–1.53)
Bird	**4.56(2.00–10.37)**	1.69 (0.84–3.38)	**3.04(1.24–7.50)**	0.58(0.28–1.19)	1.51(0.61–3.73)	1.67(0.81–3.44)	0.77(0.41–1.45)
Fish	**2.08(1.07–4.03)**	0.87(0.50–1.52)	0.43(0.13–1.39)	0.89(0.58–1.34)	1.44(0.83–2.50)	1.36(0.85–2.18)	1.02(0.69–1.50)

^a^No pet keeping was set as reference. Odds ratio was adjusted for gender, age, total income, family allergic history, home location and home dampness.

**Table 4:** Adjusted odds ratio of pet keeping for asthma and allergy among children when an avoidance behavior is adjusted^a^.

	Current wheeze	Current dry cough	Diagnosed asthma	Current rhinitis	Diagnosed rhinitis	Current eczema	Diagnosed eczema
No pet keeping (reference)	1	1	1	1	1	1	1
Pet keeping currently	**2.13(1.02–4.47)**	1.32(0.80–2.16)	**3.37(1.58–7.19)**	**1.72(1.16–2.54)**	**3.60–2.07–6.27)**	**2.53(1.57–4.08)**	**1.92(1.31–2.81)**
Cat	1.95(0.72–5.29)	1.14(0.61–2.15)	**6.34(2.75–14.65)**	**1.84(1.14–2.97)**	**4.24(2.02–8.88)**	**3.30(1.86–5.87)**	**2.10(1.35–3.28)**
Dog	1.83(0.92–3.66)	1.17(0.76–1.80)	**2.57(1.29–5.10)**	**1.73(1.24–2.40)**	**3.63(2.17–6.06)**	**2.64(1.74–4.00)**	**1.78(1.30–2.43)**
Rodent	**3.03(1.15–7.95)**	1.51(0.76–2.98)	**5.89(2.38–14.53)**	**1.75(1.00–3.07)**	**4.38(1.99–9.64)**	**3.19(1.66–6.13)**	**2.05(1.21–3.50)**
Bird	1.60(0.51–5.09)	1.10(0.53–2.26)	2.28(0.72–7.26)	**1.81(1.05–3.10)**	**4.44(2.05–9.64)**	**2.71(1.39–5.30)**	**1.94(1.16–3.23)**
Fish	1.82(0.87–3.82)	0.86(0.51–1.45)	1.53(0.65–3.60)	**1.66(1.13–2.43)**	4.00(2.32–6.88)	**3.35(2.14–5.27)**	**2.00(1.39–2.88)**
Pets keeping early	**1.96(1.18–3.25)**	**1.44(1.02–2.04)**	1.05(0.57–1.94)	1.01(0.76–1.36)	**1.26(0.83–1.91)**	1.04(0.72–1.50)	1.02(0.77–1.34)
Cat	**2.92(1.31–6.49)**	1.48(0.85–2.58)	**3.17(1.54–6.53)**	1.38(0.88–2.18)	**2.30(1.21–4.37)**	1.56(0.89–2.71)	1.15(0.75–1.77)
Dog	**1.70(1.05–2.75)**	1.19(0.88–1.62)	0.91(0.52–1.60)	1.00(0.79–1.27)	1.38(0.95–2.00)	1.31(0.98–1.75)	1.06(0.86–1.32)
Rodent	2.42(0.69–8.51)	**2.40(1.08–5.36)**	**4.54(1.61–12.85)**	0.56(0.24–1.34)	1.68(0.54–5.19)	1.63(0.68–3.93)	0.72(0.34–1.52)
Bird	**5.53(2.39–12.80)**	1.82(0.90–3.70)	**3.80(1.52–9.49)**	0.63(0.30–1.30)	1.90(0.76–4.71)	1.57(0.74–3.37)	0.76(0.40–1.44)
Fish	**2.71(1.33–5.50)**	0.92(0.52–1.64)	0.55(0.17–1.83)	0.98(0.64–1.51)	1.94(1.09–3.47)	1.48(0.91–2.42)	1.02(0.68–1.53)

^a^No pet keeping was set as reference. Odds ratio was adjusted for gender, age, total income, family allergic history, home location, home dampness and avoidance behavior.

**Fig 1:**
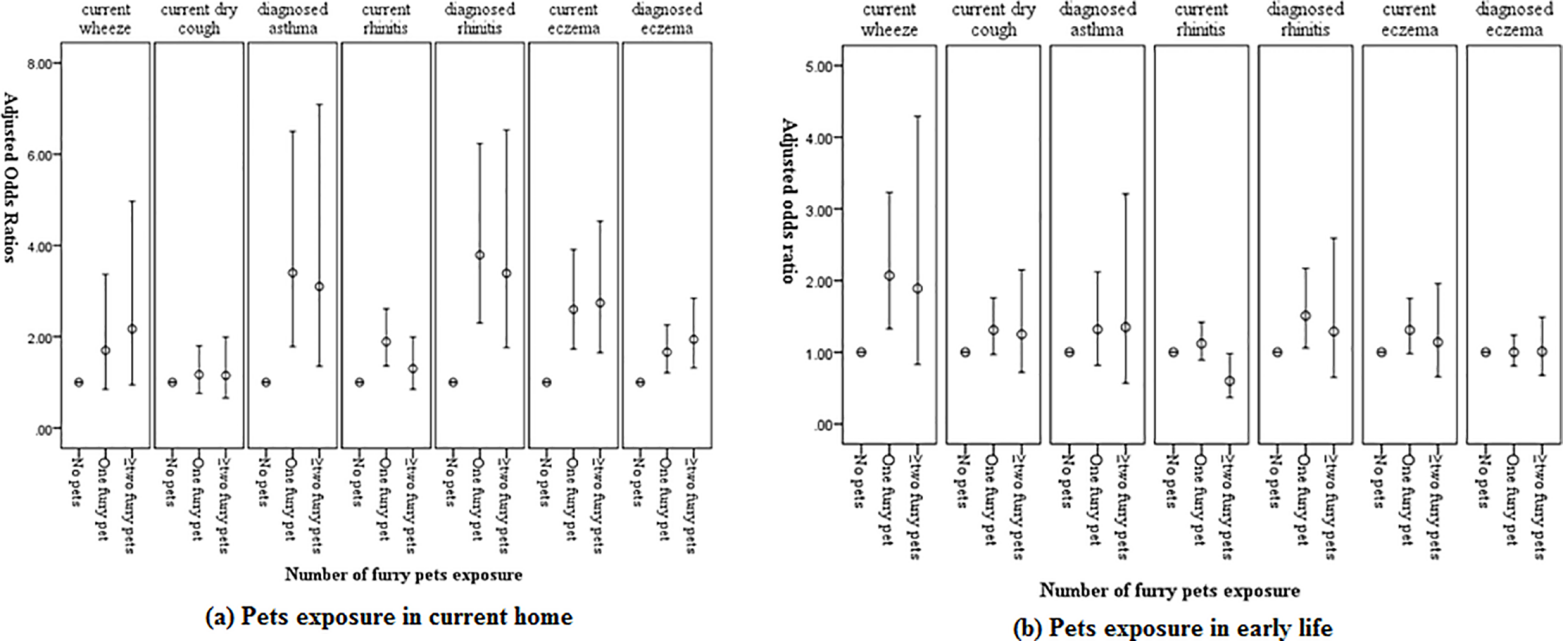
The dose-response relationship between pets keeping in home and allergies among children. ^1^Odds ratios are adjusted for gender, age, total household income, family allergic history, home location, home dampness and avoidance behavior. ^2^ Furry pet: cats, dogs, rodents and birds.

In order to explore whether there is a dose-response relationship between the number of pets and allergic diseases, furry pets including cats, dogs, rodents and birds were reclassified into two categories: one furry pet, two or more furry pets. Respectively, in current life, 12.9% families had one furry pet, while 4.5% had at least two furry pets. The dose response relationship between the number of furry pets and asthma and allergy in current home is showed in Fig 1. It indicates that pet keeping in current home have a clear dose-responses with current wheeze, current eczema and diagnosed eczema. After stratifying for rural, suburban and urban areas, we found that the dose-response relationship only exists in rural and urban areas (S2 Table).

## Discussion

Our study investigated the pet exposure in Chinese homes with a big sample size of 7366 children and a good response rate of 78%. We found that pet keeping is a risk factor for children’s health. This is consistent with the findings from the scientific literature review [14]. Most of earlier studies were based on Western populations [15–18], however, some studies from other part of Asia or China have been published in recent year [8, 19–21]. Studies either based on Western populations or based on Asian populations [4, 5, 20] revealed that there are dose-response relationships between pet exposure and allergic sensitizations. Bornehag et al [6] found that the greater number of different types of pets, the higher risk for children to have allergic symptoms. Our study confirmed the dose-response relationship of pet keeping in current homes with current eczema and diagnosed eczema, but not for pet keeping at birth.

There are studies based on western populations that support the view that keeping animals in early life of children will protect against the allergic symptoms [22–27]. In a cross-sectional study, Bornehag et al [6] showed that current pet keeping was ‘protective’, but mainly because of an avoidance behaviour. In Sweden, there was a public awareness regarding the risks of pet keeping for allergies (due to a national campaign about allergies in 1996), which can explain the high avoidance behaviour. In China and Bulgaria [18], the knowledge among the general population is probably much lower. So, most of parents know little about the risks of pet keeping for children asthma and allergies, and the percentage of people who had an avoidance behaviour was low (In this study 11.0% parents reported an avoidance behaviour towards pets, and in Bulgaria 3.3% had “got rid of” and 10.6% “refrained” from having pets, compared to Sweden 27.3% reported an avoidance behaviour).

Dander, urine and saliva from pets are potential allergens, which can be carried by pet hair, cloth, indoor furniture for several months. Through the quantitative assessment of exposure to dog (Can f 1) and cat (Fel d 1) allergens, Ingram et al [28] revealed that the high prevalence of IgE antibody to cat and dog allergens were positively associated with the presence of cat and/or dog allergens in the houses. The result in our study is consistent with finding that the presence of pet increased the risk of asthma and allergy.

### Limitations

There are some limitations in this study. Information about children’s health, pet keeping, and environment of residence in this study were collected by questionnaires, why there may be some recall bias. However, the strong associations between keeping pets, pet avoidance behaviour, and different health outcomes as shown in this study, cannot be explained by such bias. The lack of data on other contact accesses to pets’ allergens (e.g. by contacting with pets allergens outside home (like daycares, schools) or contacting with allergens transferred by visitors) is another limitation of our study. However, allergen exposure through other accesses may not change the strong association between pets keeping at home and children’s health. Identification and quantification on pet allergens (Fel d 1, Can f 1) in indoor environment should be investigated in further studies.

## Conclusions

This study assessed the relationship between pet keeping in childhood and asthma and allergy in children aged 0–8 years old. The highest prevalence of asthma and allergies was reported by people living in a city. However, pet keeping had an opposite trend. The highest rate of pet keeping was reported by people from rural areas. For all participants, pet keeping in childhood was positively associated with asthma and allergy, and the children who had pets at current home had 2–3 times higher prevalences of diagnosed asthma, rhinitis and eczema.

## Supporting information

S1 File Questionnaire (Chinese version). (DOC)

S2 File Questionnaire (English version). (DOC)

S1 Table Prevalence (%) of asthma and allergy among children with different avoidance behaviors. (DOCX)

S2 Table Adjusted odds ratio of the number of furry pets keeping and asthma and allergy among children. (DOCX)

S1 Dataset (SAV)
