# Supplementary material for: Pet keeping in childhood and asthma and allergy among children in Tianjin area, China
Source: PLoS One. 2018 May 16;13(5):e0197274. doi: 10.1371/journal.pone.0197274 (PMC5955563; doi:10.1371/journal.pone.0197274)
Supplement: S1 File — (DOC) [file pone.0197274.s001.doc]

S1 File. Questionnaire (Chinese version)


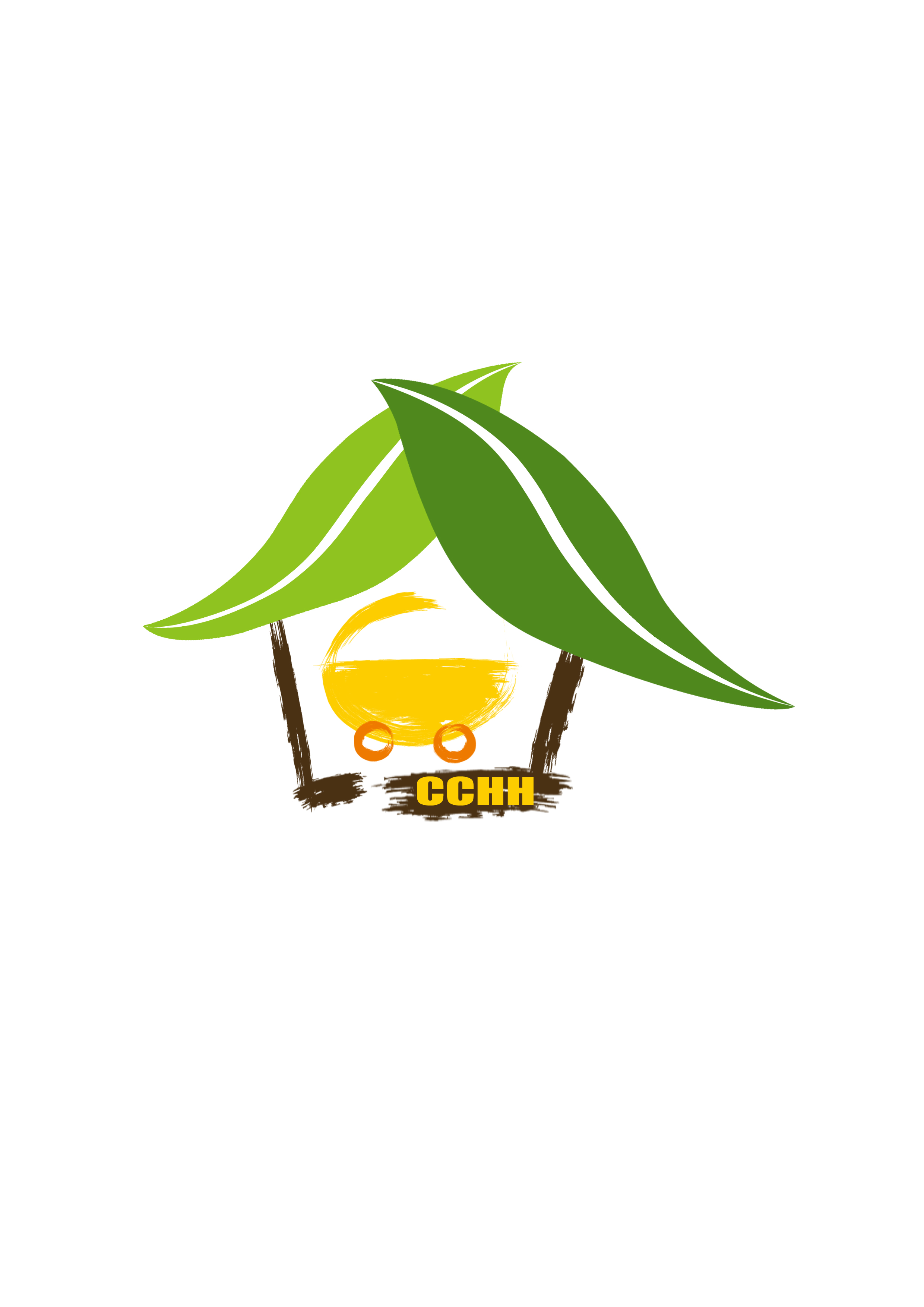
 编号：

中国室内环境与儿童健康

课题组

儿童健康与住宅空气品质研究

儿童哮喘、过敏性疾病与室内空气品质调查

天津大学

家 长 书

各位家长：

您好！

调查表明，现代人平均有超过80%的时间在室内，且65%的时间是在家里。大部分研究表明室内空气污染的程度远远高于室外环境！哪类人群是经常停留在室内并容易受到室内空气污染的严重影响呢？是儿童、孕妇、老人和慢性病病人。因此，室内空气污染对儿童的危害不容忽视！

室内装修、室内家具、杀虫喷雾剂、化妆品、厨房的油烟以及寄生于室内装修材料、生活用品和螨虫、霉菌及细菌等有害物质不仅单独对处于发育期儿童的健康产生极大的影响，它们的相互作用还会加重对儿童健康的危害。

保护孩子的健康，不仅是您的责任，也是我们科研人员的义务和责任。为此，国内多所顶尖高校、科研院所、医疗机构众多教授、博士、硕士、医生成立了中国室内环境与儿童健康课题组，涉及儿童医学、室内空气品质、环境分析等多个领域。专业的科研人员将有针对性的深入分析居住环境对儿童健康的威胁程度，研究居住环境对儿童健康的影响，保障孩子健康成长。

现在您手中这份问卷就是关于您孩子的居住环境和身体健康状况的一个调查。我们将会通过您填写的资料，分析居住环境中威胁孩子健康的潜在因素，研究影响孩子健康成长的环境因子，根据孩子的居住环境和历史健康状况对孩子未来的健康状况进行预判，有针对性的寻找减少孩子患病可能性的途径与方法。我们将以深厚的理论知识为后盾，减少您的孩子在以后生活中可能存在的健康隐患，为他们的健康成长保驾护航。本次调查采用不记名方式，调查结果统一由学校进行保密处理，数据信息仅做科研使用，所有发表的科研成果中将不会涉及您孩子和家庭的任何信息。

因此，为了您孩子的健康，我们真诚的希望，您能够百忙之中抽出时间填写这些问题，向我们真实展现您的居住环境和孩子的健康状况。我们相信，在您和我们的共同努力之下，您的孩子的明天一定会更好。

最后，再一次衷心感谢您对我们科研工作的帮助与支持！

中国室内环境与儿童健康课题组

2013年4月8日

中国-儿童-家庭-健康

儿童哮喘、过敏性疾病与室内空气品质调查

关于这项研究的详细介绍参见引言部分。如果家中1-8岁孩子超过1位，请填写年龄最小的孩子的信息。

基本资料：

孩子性别：_____ ； 出生日期：_____ 年_____ 月_____ 日；

孩子目前体重： _____ （斤）； 孩子出生时的体重：_____ （斤）；

孩子目前身高： _____ （厘米）； 孩子出生时的身高： _____ （厘米）

Ⅰ. 关于儿童和家庭背景资料

关于“孩子出生日期及常规性母乳喂养”的问题

| 1、孩子是否在预产期1周前后内出生： | □ 是  □ 比预产期提前出生，1周＜提前时间＜2周  □ 比预产期提前出生，2周＜提前时间＜3周  □ 比预产期提前出生，提前时间＞3周  □ 比预产期滞后出生，1周＜滞后时间＜2周  □ 比预产期滞后出生，2周＜滞后时间＜3周  □ 比预产期滞后出生，滞后时间＞3周  □ 不知道 |
| --- | --- |
| 2、孩子出生方式： | □自然分娩 □剖腹生产 |
| 3a、是否母乳喂养小孩(全母乳或部分母乳)： | □ 是 □ 否，如果否，跳至第4题 |
| 3b、如果是，母乳喂养持续多长时间： | □ ＜3个月 □ 3-6个月  □ 6-12 个月 □ ＞12个月 |
| 3c、如果是，有多长时间为全母乳喂养（不添加其他辅食）： | □ ＜2个月 □2-4个月  □ 5-6个月 □＞6个月 |
| 4、孩子最常使用的尿布片类型： | □ 尿布 □ 尿不湿 □ 其他 |
| 5、孩子使用什么样的奶瓶： | □ 不锈钢 □ 陶瓷 □ 玻璃  □ 塑料 □ 其他______ |
| 6a、您的孩子是否使用过安抚（安慰）奶嘴？ | □ 是 □ 否，如果否，跳至问题7 |
| 6b、如果安抚奶嘴掉到地上或沾上灰尘等脏东西，您如何清洁后放回孩子嘴里？ | □ 用嘴吮吸干净 □ 用纸/布擦拭  □ 用清水冲洗 □ 用沸水消毒  □ 使用专门的清洁用品 □ 其他_____ |

关于“儿童和家庭成员健康状况”的问题

| 7、有多少位未满9周岁的孩子长期住在家里  （包括被调研的小孩）： | □ 1位 □ 2位 □ 3位  □ 4位或更多 |
| --- | --- |
| 8、有多少位9-18周岁的青少年儿童长期住在家里： | □ 无 □ 1位 □ 2位 □ 3位  □ 4位或更多 |
| 9、有多少位18周岁以上的人长期住在家里： | □ 无 □ 1位 □ 2位 □ 3位  □ 4位或更多 |
| 10、母亲的文化教育程度： | □ 小学 □ 初中 □ 高中 □ 本科  □ 硕士 □ 博士 |
| 11、父亲的文化教育程度： | □ 小学 □ 初中 □ 高中 □ 本科  □ 硕士 □ 博士 |
| 12、母亲从事的职业： | □ 农业 □ 工业 □ 商业  □ 教育业 □ 服务业 □ 其他 _____ |
| 13、父亲从事的职业： | □ 农业 □ 工业 □ 商业  □ 教育业 □ 服务业 □ 其他 _____ |
| 14、以下哪项代表了家庭所有成员去年全年的总收入情况，包括工资收入、生意净收入、租金、养老金、股息、利息、社会保险的付款以及其他收入： | □ ＜3万人民币 □ 3-5万人民币  □ 5-10万人民币 □ 10-20万人民币  □ 20-40万人民币 □ 40-80万人民币  □80-200万人民币 □＞200万人民币 |

关于“日间托儿所”的问题

| 15、孩子在上小学之前，是待在家里还是送去日间托儿所： | □ 日间托儿所，＞20时/周  □ 日间托儿所，10-20时/周  □ 日间托儿所，＜10时/周  □ 与父母一起呆在家里  □ 与祖父母一起呆在家里  □ 由保姆在家里照看  □ 之前送去日托儿所，现在在家 |
| --- | --- |
| 16、如果曾待在日间托儿所，孩子多大的时候被送去： | □ ＜1周岁 □ 1-2周岁  □ 2-3周岁 □ 3-4周岁  □ ＞4周岁 |
| 17、孩子所在日托所是什么类型 ： | □ 私立托儿所 □ 公立托儿所 |
| 18、孩子所在托儿所的班级里总共有多少孩子： | □ ＜10个 □ 10-30个 □ ＞30个 |

Ⅱ. 关于儿童及家庭成员的健康状况

关于“儿童哮喘”的问题

| 19a、过去任何时候，孩子是否曾出现过呼吸困难，发出像哮鸣一样的声音： | □ 是  □ 否，如果否，跳至24题 |
| --- | --- |
| 19b、如果是，孩子多大的时候第一次出现这种呼吸困难的症状： | □ ＜1岁时 □ 1-2岁时 □ 3-4岁时  □ 5-6岁时 □ ＞6岁时 |
| 20a、过去12个月里，孩子是否有过呼吸困难，发出像哮鸣一样的声音： | □ 是  □ 否，如果否,跳至24题 |
| 20b、如果是，在什么情况下出现这种症状（可多选）： | □ 感冒的时候 □ 锻炼身体时  □ 笑或哭时 □ 玩耍或室外运动时 □与动物接触时 □ 其他________ |
| 21、过去12个月里，有几次出现上述呼吸困难的问题？ | □ 从来没有 □ 1-3次 □ 4-12次  □ ＞12次 |
| 22、过去12个月里，上述呼吸困难问题是否干扰了孩子的晚间睡眠？ | □ 从来没有 □ 平均而言，＜1周1次  □ 平均而言，≥1周1次 |
| 23、过去12个月里，上述呼吸困难问题是否严重到限制了孩子的言语，比如：每次呼吸间隔只能说出1-2个词？ | □ 是  □ 否 |
| 24、过去12个月里，在没有感冒或胸腔感染的情况下，孩子是否有夜晚干咳超过两周的现象： | □ 是  □ 否 |
| 25、是否由于上述呼吸困难或夜间干咳问题，带您的孩子去看医生？ | □ 是  □ 否 |
| 26、孩子是否被医生确诊过患有哮喘： | □ 是 □ 否 |
| 27、孩子是否患过喉炎（呼吸困难且伴有犬哮一样的咳嗽）： | □ 是  □ 否 |
| 28、孩子是否患过肺结核？ | □ 是 □ 否 |
| 29a、您的孩子是否被医生确诊过患有肺炎： | □ 是,  □ 否，如果否，跳至30a题 |
| 29b、如果是，第一次患肺炎孩子多大？ | _____ _岁 |
| 29c、如果是，目前为止总共患过几次： | □ 只有1次 □ 2-3次 □ 4次及以上 |

关于“儿童鼻炎”的问题

| 30a、过去任何时候，孩子在没有感冒的情况下是否曾有打喷嚏、鼻塞、流鼻涕的问题： | □ 是  □ 否，如果否，跳至34题 |
| --- | --- |
| 30b、如果是，第一次出现这种情况是在孩子多大的时候： | □ 1岁以前 □ 1-2岁 □ 3-4岁  □ 5-6岁 □ 6岁以后 |
| 31a、过去12个月里，您的孩子在没有感冒的情况下是否有打喷嚏、鼻塞、流鼻涕的问题： | □ 是  □ 否，如果否，跳至34题 |
| 31b、过去12个月里，上述打喷嚏、鼻塞、流鼻涕的问题出现在哪些月份(可多选)？ | □1月 □ 2月 □ 3月 □ 4月  □5月 □ 6月 □ 7月 □ 8月  □9月 □ 10月 □ 11月 □ 12月 |
| 32、过去12个月里，上述打喷嚏、鼻塞、流鼻涕的问题多大程度上干扰了孩子的日常活动？ | □ 从来没有 □ 稍微，有一些  □ 中等程度 □ 很大程度 |
| 33、过去12个月里，上述打喷嚏、鼻塞、流鼻涕的问题是否伴随有眼睛刺痒、流眼泪的现象？ | □是  □否 |
| 34、孩子是否被诊断出患有花粉症或过敏性鼻炎： | □ 是 □ 否 |
| 35a、过去12个月里，孩子感冒过几次： | □ 从来没有 □ 1-2次 □ 3-5 次  □ 6-10次 □ ＞10次 □ 不知道 |
| 35b、孩子感冒通常出现在哪些季节（可多选）： | - 春 □ 夏 □ 秋 □ 冬 |
| 35c、孩子一次感冒通常持续多久： | □ ＜2周 □ 2-4周 □ ＞4周 |
| 36、您的孩子是否患过耳炎： | □ 否 □ 是，1-2次  □ 是，3-5次 □是，＞5次 |

关于“儿童湿疹”的问题

| 37a、过去任何时候，孩子是否出现过湿疹症状，如皮肤发红、刺痒，出现皮疹，继之皮肤发糙、脱屑或出现水泡，脂水渗溢： | □ 是  □ 否，如果否,跳至39题 |
| --- | --- |
| 37b、如果是，第一次出现这种情况是在孩子多大的时候： | □ 1岁以前 □ 1-2岁 □ 3-4岁  □ 5-6岁 □ 6岁以后 |
| 37c、 如果是，上述皮肤问题是否出现在肘关节，膝关节，踝关节，臀部，面部等部位？ | - 是   □ 否 |
| 38a、过去12个月里，孩子是否有上述皮肤问题： | □ 是 □ 否，如果否，跳至39题 |
| 38b、过去12个月里，孩子是否由于上述皮肤问题而夜晚无法入睡： | - 从未 □每周不到一次 - 每周一次或更多 |
| 39、孩子是否被医生确诊过患有湿疹： | □ 是 □ 否 |

关于“儿童对食物的反应”的问题

| 40a、是否有过食物引起的湿疹、荨麻疹、腹泻、嘴唇或眼睛肿胀等过敏症状： | □ 是 □ 否  □ 不知道 |
| --- | --- |
| 40b、如果是，以下哪种食物引起 (可多选) ： | □ 牛奶或奶制品 □ 鸡蛋 □ 鱼 □ 花生  □ 坚果类，杏仁 □ 海产品类  □ 蔬菜，如番茄、土豆、芹菜等  □ 面粉（如小麦、大麦、黑麦、荞麦等）  □ 豆类（如大豆、豌豆等）  □ 水果（如苹果、芒果等）  □ 其他食物 |

关于“儿童使用抗生素治疗”问题

| 41a、孩子是否接受过抗生素治疗，如青霉素等(可多选)： | □ 否,从未 □ 是，0-12个月时 □ 是，12-24个月时 □ 是，比24个月大时 |
| --- | --- |
| 41b、若为是，使用抗生素的原因为： | __________________ |
| 42、如果孩子在0-12个月时接受过抗生素治疗，那么共接受过几次： | □ 1次 □ 2次 □ ≥3次 |

关于儿童慢性“现代性”疾病的问题

43、孩子是否被确诊过患有以下疾病？

|  | 是否由医生诊断出？ | | 是否使用药物？ | | 什么时候被确诊？ |
| --- | --- | --- | --- | --- | --- |
|  | 是 | 否 | 是 | 否 |
| 糖尿病 |  |  |  |  |  |
| 轻微脑功能障碍 |  |  |  |  |  |
| 注意力缺陷多动障碍 |  |  |  |  |  |
| 自闭症 |  |  |  |  |  |
| 阿斯伯格综合症 ^1^ |  |  |  |  |  |
| 抽动秽语综合征 ^2^ |  |  |  |  |  |
| 隐睾症、尿道下裂 |  |  |  |  |  |

注：1.阿斯伯格综合症，是一种主要以社会交往困难，局限而异常的兴趣行为模式为特征的神经系统发育障碍性疾病；相较于其他泛自闭症障碍，仍相对保有语言及认知发展。阿斯伯格症患者经常出现肢体互动障碍和语言表达方式异常等状况，但并不需要接受治疗。

2. 抽动秽语综合征，也称妥瑞氏症，是指以不自主的突然的多发性抽动以及在抽动的同时伴有暴发性发声和秽语为主要表现的抽动障碍。

关于“家庭其他成员健康”的问题

| 44a、家庭成员中是否存在哮喘或过敏性问题： | □ 是 □ 否，如果否，跳至45题 |
| --- | --- |
| 44b、如果“是”，请问是来自哪位家庭成员的什么样的问题(可多选)： | 父亲： □ 哮喘 □ 鼻炎 □ 湿疹  母亲： □ 哮喘 □ 鼻炎 □ 湿疹  孩子的兄弟姐妹：  □ 哮喘 □ 鼻炎 □ 湿疹 |
| 45、在过去12个月里，家庭成员各感冒过几次： | 父亲：□ 0次 □ 1-2次 □ 3-4次  □ ≥5次 □ 不知道/不适用  母亲：□ 0次 □ 1-2次 □ 3-4次  □ ≥5次 □ 不知道/不适用  兄弟姐妹：  □ 0次 □ 1-2次 □ 3-5次 □ 6-10次  □＞10次 □ 不知道/不适用 |

关于“孩子父亲或母亲健康”的问题

| 46、请问如下47题是由谁来回答的： | | □ 母亲 □ 父亲 □ 其他 ______ | |  |
| --- | --- | --- | --- | --- |
| 47、在过去的3个月里您是否有以下症状（请对所有问题做出答复）： | | | |  |
| 疲劳，乏力： | - 是，经常（每周） - 是，有时 □ 否，从不 | | 如果是，您认为是否和居住环境有关：□ 是 □ 否 | |
| 感觉头重： | - 是，经常（每周） - 是，有时 □ 否，从不 | | 如果是，您认为是否和居住环境有关：□ 是 □ 否 | |
| 头痛： | - 是，经常（每周） - 是，有时 □ 否，从不 | | 如果是，您认为是否和居住环境有关：□ 是 □ 否 | |
| 恶心，眩晕： | - 是，经常（每周） - 是，有时 □ 否，从不 | | 如果是，您认为是否和居住环境有关：□ 是 □ 否 | |
| 注意力难以集中： | - 是，经常（每周） - 是，有时 □ 否，从不 | | 如果是，您认为是否和居住环境有关：□ 是 □ 否 | |
| 眼睛发痒、有灼烧或刺痛感： | - 是，经常（每周） - 是，有时 □ 否，从不 | | 如果是，您认为是否和居住环境有关：□ 是 □ 否 | |
| 鼻子刺痛、堵塞或流鼻涕： | - 是，经常（每周） - 是，有时 □ 否，从不 | | 如果是，您认为是否和居住环境有关：□ 是 □ 否 | |
| 喉咙嘶哑、干燥： | - 是，经常（每周） - 是，有时 □ 否，从不 | | 如果是，您认为是否和居住环境有关：□ 是 □ 否 | |
| 咳嗽： | - 是，经常（每周） - 是，有时 □ 否，从不 | | 如果是，您认为是否和居住环境有关：□ 是 □ 否 | |
| 面部皮肤干燥、红晕： | - 是，经常（每周） - 是，有时 □ 否，从不 | | 如果是，您认为是否和居住环境有关：□ 是 □ 否 | |
| 头皮或耳朵起屑刺痒： | - 是，经常（每周） - 是，有时 □ 否，从不 | | 如果是，您认为是否和居住环境有关：□ 是 □ 否 | |
| 手部皮肤干燥、发痒、发红： | - 是，经常（每周） - 是，有时 □ 否，从不 | | 如果是，您认为是否和居住环境有关：□ 是 □ 否 | |

Ⅲ. 关于儿童的居住环境状况

这章的大部分问题是关于儿童主要住处情况，即孩子多数时间的居住场所。如果孩子目前跟爷爷、奶奶（或姥姥、姥爷）住在一起，请回答孩子爷爷、奶奶（或姥姥、老爷）的住处情况。同时，我们也想了解孩子出生后早期居住地的环境情况。

| 48、 自从孩子出生后，孩子是否一直居住在目前住处： | □ 是  □ 否（若为否，居住时期开始于哪一年？___） |
| --- | --- |
| 49a、 孩子是否每个月有超过10天在其他住处住宿： | □ 是  □ 否 |
| 49b、如果是，孩子跟谁一起居住： | __________ |

关于“孩子目前住处的周围环境”的问题

| 50、您的住处位于哪里： | □ 城市中心 □ 郊区 □ 农村 □ 其他 |
| --- | --- |
| 51、住处是否临近高速公路或交通干线： | □ 是 □ 否 |
| 52、住处是否临近牲畜养殖地（如奶牛、猪、马等） | □ 是  □ 否 |

关于“孩子目前住处建筑类型”的问题

| 53、目前孩子住在什么样的房子中： | □平顶平房 □坡顶平房 □ 别墅或排房  □多层楼房（7层及以下）  □ 高层楼房（7层以上） |  |
| --- | --- | --- |
| 54、请估算房子大小(平方米)： | □ ≤40 □ 41-60 □ 61-75  □ 76-100 □ 101-150 □ ＞150 |  |
| 55、请估算房龄（年）： | □ ＜10 □ 10-20 □ 20-30  □ 30-40 □ 40-50 □ ＞50 □ 不知道 | |
| 56、现有住宅墙体外有没有保温层： | □ 有 □ 没有 □ 不知道 | |
| 57、您现在的住处是租赁的吗： | □ 是 □ 否 | |

关于“孩子的房间”的问题

（所谓孩子的房间是指该儿童日夜大部分时间活动的场所）

| 58、孩子大部分时间睡在哪个房间（只能选一项）： | □ 孩子自己的房间 □ 和兄弟姐妹分享房间  □ 和父母或祖父母睡一个房间  □ 其他_______ |
| --- | --- |
| 59、孩子房间的朝向？ | □ 东 □ 西 □ 南 □ 北  □ 其他_____ |

关于“目前住处内部装修”的问题

(请注意，油毡地板较硬，不能用针刺透；而PVC地板较软，能刺透。)

| 60、 不同功能房间铺设地板的材料： | 孩子的房间 | □ 油毡地板 □（PVC）聚氯乙烯 □ 地毯  □ 纯木地板 □ 复合木地板 □ 水泥地板  □ 石头/瓷砖□ 其他/不知道 |
| --- | --- | --- |
|  | 父母的房间 | □ 油毡地板 □（PVC）聚氯乙烯 □ 地毯  □ 纯木地板 □ 复合木地板 □ 水泥地板  □ 石头/瓷砖□ 其他/不知道 |
|  | 客厅 | □ 油毡地板 □（PVC）聚氯乙烯 □ 地毯  □ 纯木地板 □ 复合木地板 □ 水泥地板  □ 石头/瓷砖□ 其他/不知道 |
|  | 厨房 | □ 油毡地板 □（PVC）聚氯乙烯 □ 地毯  □ 纯木地板 □ 复合木地板 □ 水泥地板  □ 石头/瓷砖□ 其他/不知道 |
| 61a、孩子睡觉的房间的墙面材料（可多选）： | | □ 墙面漆，涂料 □ 乳胶漆 □ 壁纸  □石头/瓷砖 □ 木质板 □ 纺织品  □ 石灰（大白） □ 其他 |
| 61b、孩子睡觉的房间的窗户类型： | | □ 木框 □ 铝框 □ 塑钢窗 |
| 61c、孩子睡觉的房间窗户的玻璃类型： | | □ 单层玻璃 □双层玻璃 □双层充气玻璃 |
| 62a、孩子父母房间的墙面材料（可多选）： | | □ 墙面漆，涂料 □ 乳胶漆 □ 壁纸  □石头/瓷砖 □ 木质板 □ 纺织品  □ 石灰（大白） □ 其他 |
| 62b、孩子父母房间的窗户类型： | | □ 木框 □ 铝框 □ 塑钢窗 |
| 62c、孩子父母房间窗户的玻璃类型： | | □ 单层玻璃 □双层玻璃 □双层充气玻璃 |

关于“目前住处采暖及通风”的问题

| 63a、冬季住所采用什么样的采暖形式： | □电暖器 □ 暖气片 □ 地板采暖  □ 空调采暖 □ 煤炉 □ 火炕  □ 其他 □ 无供暖 |
| --- | --- |
| 63b、冬季您住处的舒适度如何： | □ 正合适 □ 太热 □ 太冷 |
| 64a、夏季住所采用什么样的降温方式： | - 空调 □ 风扇 □ 开窗通风 □ 其他 |
| 64b、夏季您住处的舒适度如何： | □ 正合适 □ 太热 □ 太冷 |
| 65、住宅通风系统（可多选）？ | □ 开门窗通风，住宅内没有排风扇  □ 开门窗通风，并且厨房有排风扇（口）  □ 开门窗通风，并且厕所有排风扇（口）  □ 开门窗通风，并且卧室有排（送）风扇  □ 机械通风 □ 其他 |
| 66、您一般采用哪种燃料做饭： | □ 煤 □ 秸秆/木材 □ 天然气  □ 电 □ 其它 |

关于“对目前住处翻新修整和扩展”的问题

| 67a、是否对您的住所进行过翻新和扩展： | | □ 是  □ 否，跳至68题  □ 不知道，跳至68题 | |
| --- | --- | --- | --- |
| 67b、如果是，什么时候对其进行的翻新或修整： | | □ 1-2年以前 □ 3-4年以前  □ 5-6年以前 □ 7-8年以前  □ 9-10年以前 | |
| 67c、如果是，是否是由于房子出现潮湿和发霉的问题： | | □ 是 □ 否  □ 不知道 | |
| 68、对于下列房间，孩子出生前6个月或孩子出生后1年内是否更换过房间的地板材料： | 孩子的房间 | □ 是 □ 否 □ 不知道 |  |
|  | 父母的房间 | □ 是 □ 否 □ 不知道 |  |
|  | 其他房间 | □ 是 □ 否 □ 不知道 |  |
| 69、对于下列房间，孩子出生前6个月或孩子出生后1年内是否对该房间重新粉刷过： | 孩子的房间 | □ 是 □ 否 □ 不知道 |  |
|  | 父母的房间 | □ 是 □ 否 □ 不知道 |  |
|  | 其他房间 | □ 是 □ 否 □ 不知道 |  |

关于“目前住处是否潮湿”的问题

| 70、下列房间的地板、墙面及天花板是否有可见的发霉迹象： | 孩子的房间 | □ 是 □ 否 □ 不知道 |
| --- | --- | --- |
|  | 父母的房间 | □ 是 □ 否 □ 不知道 |
|  | 其他房间 | □ 是 □ 否 □ 不知道 |
|  | 浴室 | □ 是 □ 否 □ 不知道 |
| 71、下列房间的地板、墙面及天花板是否有可见的由于潮湿而造成的污斑或褪色： | 孩子的房间 | □ 是 □ 否 □ 不知道 |
|  | 父母的房间 | □ 是 □ 否 □ 不知道 |
|  | 其他房间 | □ 是 □ 否 □ 不知道 |
|  | 浴室 | □ 是 □ 否 □ 不知道 |
| 72、您是否怀疑您住处的地板内、墙及天花板内存在潮湿发霉的问题，即便房间表面没有明显的迹象： | | □ 是 □ 否 □ 不知道 |
| 73、下列房间的地板材料是否有褪色、变黑或剥离的迹象： | 孩子的房间 | □ 是 □ 否 □ 不知道 |
|  | 父母的房间 | □ 是 □ 否 □ 不知道 |
|  | 其他房间 | □ 是 □ 否 □ 不知道 |
|  | 浴室 | □ 是 □ 否 □ 不知道 |
| 74、对于下列房间，是否有过水管爆裂等造成的水损问题： | 孩子的房间 | □ 是 □ 否 □ 不知道 |
|  | 父母的房间 | □ 是 □ 否 □ 不知道 |
|  | 其他房间 | □ 是 □ 否 □ 不知道 |
|  | 浴室 | □ 是 □ 否 □ 不知道 |
| 75、对于下列房间，冬季时，在窗户的内侧底部是否有凝水现象： | 孩子的房间 | □ 否，从来没有过 □ 是，高度＜5厘米  □ 是，5-25厘米 □ 是，＞25厘米 □ 不知道 |
|  | 父母的房间 | □ 否，从来没有过 □ 是，高度＜5厘米  □ 是，5-25厘米 □ 是，＞25厘米 □ 不知道 |
|  | 客厅 | □ 否，从来没有过 □ 是，高度＜5厘米  □ 是，5-25厘米 □ 是，＞25厘米 □ 不知道 |

关于“目前住处是否有异味”的问题

| 76a、下面气味问题（76b）由谁回答？ | | □ 父亲 □ 母亲 □ 其他人________ |
| --- | --- | --- |
| 76b、在过去的3个月里，您是否被住所内的下述任何（一种或多种）气味所烦扰： | 通风不良引起的不新鲜气味 | □ 是，经常（每周） □ 是，有时  □ 否，从来没有 |
|  | 令人不愉快的气味 | □ 是，经常（每周） □ 是，有时  □ 否，从来没有 |
|  | 辛辣刺鼻的气味 | □ 是，经常（每周） □ 是，有时  □ 否，从来没有 |
|  | 发霉的气味 | □ 是，经常（每周） □ 是，有时  □ 否，从来没有 |
|  | 烟草的气味 | □ 是，经常（每周） □ 是，有时  □ 否，从来没有 |
|  | 感觉空气干燥 | □ 是，经常（每周） □ 是，有时  □ 否，从来没有 |
|  | 感觉空气潮湿 | □ 是，经常（每周） □ 是，有时  □ 否，从来没有 |

关于“孩子出生后早期居住地”的问题

（若孩子出生后一直住在目前的住宅内，请跳过问题77，78，79；若孩子出生后曾换过住宅，请回答问题77，78，79，对孩子出生后早期居住地的环境进行描述。）

| 77、 孩子房间的地板覆盖材料： | | □ 油毡地板 □（PVC）聚氯乙烯 □ 地毯  □ 纯木地板 □ 复合木地板 □ 水泥地板  □ 石头/瓷砖 □ 其他/不知道 |
| --- | --- | --- |
| 78、在孩子早期居住的房间内，您是否注意到： | 房间地板、墙面及天花板有可见的发霉或潮湿的迹象 | □ 是，经常（每周） □ 是，有时  □ 否，从来没有 |
|  | 房间的地板材料有褪色、变黑或剥离的迹象 | □ 是，经常（每周） □ 是，有时  □ 否，从来没有 |
|  | 房间有过水管爆裂等造成的水损问题 | □ 是，经常（每周） □ 是，有时  □ 否，从来没有 |
|  | 房间窗户的内侧底部有凝水或水汽现象： | □ 是，经常（每周） □ 是，有时  □ 否，从来没有 |
|  | 怀疑地板内、墙及天花板内存在潮湿发霉的问题，即便房间表面没有明显的迹象 | □ 是，经常（每周） □ 是，有时  □ 否，从来没有 |
| 79、在孩子早期居住的房间内，是否出现过下述任何（一种或多种）气味： | 通风不良引起的不新鲜气味 | □ 是 □ 否 |
|  | 令人不愉快的气味 | □ 是 □ 否 |
|  | 辛辣刺鼻的气味 | □ 是 □ 否 |
|  | 发霉的气味 | □ 是 □ 否 |
|  | 烟草的气味 | □ 是 □ 否 |
|  | 感觉空气干燥 | □ 是 □ 否 |
|  | 感觉空气潮湿 | □ 是 □ 否 |

关于“动物饲养与接触”的问题

| 80a、目前，您的住处是否饲养动物或宠物： | □ 是  □ 否，如果否，跳至81a题 |
| --- | --- |
| 80b、如果是，您饲养什么动物，共几只： | □ 猫 □ 狗  □ 啮齿目动物（如兔子、仓鼠、天竺鼠等）  □ 鸟类 □ 鱼、爬行动物  □ 其他毛皮类动物 |
| 81a、在孩子1岁期间，是否饲养动物或宠物： | □ 是  □ 否，如果否，跳至82题 |
| 81b、如果是，您饲养什么动物，共几只： | □ 猫 □ 狗  □ 啮齿目动物（如兔子、仓鼠、天竺鼠等）  □ 鸟类 □ 鱼、爬行动物  □ 其他毛皮类动物 |
| 82、您是否由于家庭成员的过敏性疾病而放弃继续饲养某些动物/宠物： | □ 是  □ 否 |
| 83、您是否由于家庭成员的过敏性疾病而拒绝或克制饲养某些动物/宠物： | □ 是  □ 否 |
| 84、孩子1岁期间是否经常接触到牲畜（如：牛、马、羊、猪等）： | □ 是  □ 否 |
| 85、母亲怀孕期间是否经常接触到牲畜（如：牛、马、羊、猪等）： | □ 是  □ 否 |

关于“住处清洁”的问题

| 86a、您多长时间对孩子的房间清扫一次： | | □ 每天 □一周两次 □ 一周一次  □ 两周一次 □ 一月一次 □ 很少 |  |
| --- | --- | --- | --- |
| 86b、您是否由于家庭成员的过敏性疾病而改变过日常清洁习惯： | | □ 是 □ 否  □ 不知道 |  |
| 86c、您清洁孩子房间地面的方法(可多选)： | | □ 用扫帚扫 □用拖把  □ 用吸尘器 □ 其他______ |  |
| 86d、您清洁地面时是否使用清洁剂： | | □ 是 □ 否 |  |
| 87、您平均多久开窗通风一次： | | □ 每天 □一周两次 □ 一周一次  □ 两周一次 □一月一次 □ 很少 |  |
| 88、您多久晒一次被褥： | | □ 经常 □ 有时 □ 从不 |  |
| 89、您或您的家人使用以下几种产品的频率： | 加湿器 | □ 每天 □ 一周几次 □ 一月几次  □ 一年几次 □ 更少 □ 从来不用 | |
|  | 离子发生器 | □ 每天 □ 一周几次 □ 一月几次  □ 一年几次 □更少 □从来不用 | |
|  | 臭氧发生器 | □ 每天 □ 一周几次 □ 一月几次  □ 一年几次 □ 更少 □从来不用 | |
|  | 空气净化器 | □ 每天 □ 一周几次 □ 一月几次  □ 一年几次 □ 更少 □从来不用 | |
|  | 空气清新剂 | □ 每天 □ 一周几次 □ 一月几次  □ 一年几次 □ 更少 □从来不用 | |
|  | 厨房清洁剂（洗洁精，除污剂） | □ 每天 □ 一周几次 □ 一月几次  □ 一年几次 □ 更少 □从来不用 | |
|  | 浴室或洗衣清洁剂（洗衣液、马桶液） | □ 每天 □ 一周几次 □ 一月几次  □ 一年几次 □ 更少 □从来不用 | |
|  | 家具清洁产品（如抛光，打蜡） | □ 每天 □ 一周几次 □ 一月几次  □ 一年几次 □ 更少 □从来不用 | |
|  | 杀虫剂 | □ 每天 □ 一周几次 □ 一月几次  □ 一年几次 □ 更少 □从来不用 | |
|  | 燃烧蜡烛或焚香 | □ 每天 □ 一周几次 □ 一月几次  □ 一年几次 □ 更少 □从来不用 | |

关于“家庭成员吸烟”的问题

| 90a、家庭成员中是否有人吸烟（住在一起的）： | | □ 是，母亲 □ 是，父亲  □ 是，兄弟姐妹 □ 是，其他人  □ 否，如果否，跳至91题 |  |
| --- | --- | --- | --- |
| 90b、如果是，他们一般会在住处的什么地方吸烟（可多选）： | | - 阳台或平台 - 居室内 |  |
| 90c、如果是在居室里，他们总共每天吸烟多少支： | | - ＜10支/日 □ 10-20支/日 - ＞20支/日 □ 不知道 |  |
| 91、孩子1岁期间父母亲中谁有吸烟的习惯： | 母亲 | □ 是 □ 否 | |
|  | 父亲 | □ 是 □ 否 | |
| 92、怀孕期间父母亲中谁有吸烟的习惯： | 母亲 | □ 是 □ 否 | |
|  | 父亲 | □ 是 □ 否 | |

关于“孩子床上用品”的问题

|  | **目前** | **1岁时** |
| --- | --- | --- |
| 93、孩子的枕头（多选） | **□** 海绵枕 | **□** 海绵枕 |
|  | **□** 荞麦枕 | **□** 荞麦枕 |
|  | **□** 羽毛枕 | **□** 羽毛枕 |
|  | **□** 其他______ | **□** 其他______ |
|  | **目前** | **1岁时** |
| 94、孩子的被子填充物（多选） | **□** 棉花 | **□** 棉花 |
|  | **□** 纤维棉 | **□** 纤维棉 |
|  | **□** 羽毛被 | **□** 羽毛被 |
|  | **□** 其他______ | **□** 其他______ |

Ⅳ关于“饮食习惯”的问题

媒体经常会暗示我们的生活习惯会造成一些过敏性疾病，但是还没有数据证明这一设想，因此我们希望通过以下问题找到关于我们现代生活方式与饮食习惯之间的关系。

| 95、过去12个月里， 平均而言，孩子食用如下食物和饮料的频率： | |
| --- | --- |
| 肉类（如猪、牛、羊、鸡肉等） | □ 很少，偶尔 □ 每周1-2次 □ 每周3-4次  □ 每周5-6次 □ 每天1次或更多 |
| 海鲜（鱼、贝等） | □ 很少，偶尔 □ 每周1-2次 □ 每周3-4次  □ 每周5-6次 □ 每天1次或更多 |
| 水果 | □ 很少，偶尔 □ 每周1-2次 □ 每周3-4次  □ 每周5-6次 □ 每天1次或更多 |
| 蔬菜 | □ 很少，偶尔 □ 每周1-2次 □ 每周3-4次  □ 每周5-6次 □ 每天1次或更多 |
| 豆类或谷物类（小米，黄豆等） | □ 很少，偶尔 □ 每周1-2次 □ 每周3-4次  □ 每周5-6次 □ 每天1次或更多 |
| 大米白面类（面条，馒头，面包等） | □ 很少，偶尔 □ 每周1-2次 □ 每周3-4次  □ 每周5-6次 □ 每天1次或更多 |
| 鸡蛋 | □ 很少，偶尔 □ 每周1-2次 □ 每周3-4次  □ 每周5-6次 □ 每天1次或更多 |
| 牛奶 | □ 很少，偶尔 □ 每周1-2次 □ 每周3-4次  □ 每周5-6次 □ 每天1次或更多 |
| 快餐类（如KFC等食品） | □很 少，偶尔 □ 每周1-2次 □ 每周3-4次  □ 每周5-6次 □ 每天1次或更多 |

关于“孩子户外活动和电脑电视使用”的问题

| 96、孩子平均每天看电视或玩电脑的时间： | □ <1小时/天 □ 1-3小时/天  □ 3-5小时/天 □ >5小时/天 |
| --- | --- |
| 97、孩子课余时间，每周户外活动的频率： | □ 很少，偶尔  □ 1-2次/周  □ 3次或更多/周 |
| 98、谁填写的这份调查问卷： | □ 爸爸 □ 妈妈 □ 其他人____ _ |
| 99、问卷完成时间 | ____ _年____ _月____ _日 |

*您的意见或建议：____________________________*

***关于第二阶段的入户调查***

***（见下页）***

***关于第二阶段的入户调查***

为了进一步确定您的居室环境对孩子健康的影响，我们希望您能同意我们对您的住所进行空气品质免费测试，并愿意带您的孩子到我们指定的医院做免费体检。

您是否愿意我们对您的房间做免费的检查？

**□**是 **□**否

您是否愿意带您的孩子到医院做免费的体检？

**□**是 **□**否

如果是，请将以下信息栏补充完整，以方便我们后期与您联系。

| 手机： |  |
| --- | --- |
| 家庭电话： |  |
| 邮箱： |  |
| 家庭地址： |  |

***谢谢您的参与！***
