# Supplementary material for: Pet keeping in childhood and asthma and allergy among children in Tianjin area, China
Source: PLoS One. 2018 May 16;13(5):e0197274. doi: 10.1371/journal.pone.0197274 (PMC5955563; doi:10.1371/journal.pone.0197274)
Supplement: S2 File — (DOC) [file pone.0197274.s002.doc]

**S2 File. Questionnaire (English version)**

ID _______

# China - Child - Home - Health

A survey of the significance of the home environment for asthma and allergies among young children

A description of the study is included in the enclosed introduction letter.

If there are more than one child 0-8 years old, please fill in the information of the youngest one.

Child’s gender: ______

Childs’s date of birth: (year)____(month)_____(date)______

Child current weithght_______Jin

Child current length________cm

Child’s weight at birth ________ Jin

Child’s length at birth _________cm

1. Background information of the child and the family

Questions concerning the date of birth of the child and the breast-feeding routines

1. Was your child born within 1 weeks of the calculated date of birth?

- Yes
- No, more than 1 but less than 2 weeks early
- No, more than 2 but less than 3 weeks early
- No, more than 3 weeks early
- No, more than 1 but less than 2 weeks late
- No, more than 2 but less than 3 weeks late
- No, more than 3 weeks late
- Not known

1. In which way the child was given birth?

- Natural delivery
- Caesarean section

3a. Was your child ever breast-fed totally or partly?

- Yes
- No

3b. If yes, for how long?

- Less than 3 months
- 3 – 6 months
- 6-12 months
- More than one year

3c. If yes, for how long was your child breast fed without adding other foods or juices?

- Less than 2 months
- 2-4 months
- 5-6 months
- More than 6 months

4 What kind of napkins (Am. diapers) is most commonly used?

- Nappy
- Disposable nappy
- Other kind

1. What kind of milk bottle does your child use?

- Stainless steel
- Ceramics
- Glass
- Plastics
- Other_______

6a. Has your child had used pacifier?

- Yes
- No

6b. If yes, when pacifier drops down on floor or is polluted by dust, how do you clean it?

- Sucking in moth
- Wipe by paper or cloth
- Rinse by clean water
- Boiling it
- Clean by cleaning product
- Other______

## Questions concerning the family and the household

7.How many children, who have not yet turned 9, are permanently living at home (including the investigated one)?

- 1 child
- 2 children
- 3 children
- 4 or more children

1. How many children / adolescents, age 9 to 18, are permanently living at home?

- None
- 1 person
- 2 persons
- 3 persons
- 4 or more persons

1. How many persons, above the age 18, are permanently living at home?

- 1 adult
- 2 adults
- 3 adults
- 4 or more adults

1. Education level of mother

- Primary school
- Junior middle school
- Senior middle school
- Bachelor
- Master
- PhD

1. Education level of father

- Primary school
- Junior middle school
- Senior middle school
- Bachelor
- Master
- PhD

1. Occupation of mother

- Agriculture
- Industry
- Commercial and Business
- Education
- Service
- Other_________

1. Occupation of father

- Agriculture
- Industry
- Commercial and Business
- Education
- Service
- Other_________

14. Which category represents the total combined income of all members of this FAMILY during the past 12 months? *This includes money from jobs, net income from business, farm or rent, pensions, dividends, interest, social security payments and any other money income received (by members of this FAMILY who are 15 years of age or older.)*

- Less than 30,000 RMB
- 30,000-50,000 RMB
- 50,000-100,000 RMB
- 100,000-200,000 RMB
- 200,000-400,000 RMB
- 400,000-800,000 RMB
- 800,000-2,000,000 RMB
- More than 2,000,000 RMB

Questions concerning day nursery, if applicable

15.Is the child staying at home or attending day nursery?

- Attending day nursery, >20 hours per week
- Attending day nursery, 10-20 hours per week
- Attending day nursery, <10 hours per week
- Staying at home with parents
- Staying at home with grandparents
- Taking care by nanny or others at home
- Attending day nursery before, but now at home

1. If the child has stayed at day nursery, at what age did the child start to attend it?

- Younger than 1 year
- 1-2 years of age
- 2-3 years of age
- 3-4 years of age
- Older than 4 years of age

1. What kind of day nursery does the child attend?

- Private day nursery
- Public day nursery

1. How many children are taken care together with your child?

- <10 children
- 10-30 children
- >30 children

1. The child’s and the family’s health

Questions concerning breathing difficulties for the child

19a). Has your child ever had wheezing or whistling in the chest at any time in the past?

- Yes
- No. If No, skip to question 24

19b). If Yes, at what age did the problem first occur?

- Prior to 1 year of age
- At 1-2 years of age
- At 3-4 years of age
- At 5-6 years of age
- Past 6 years of age

20a) Has your child had wheezing or whistling in the chest in the past 12 months?

- Yes
- No. If No, skip to question 24

20b) If Yes, under which circumstances?

More than one alternative possible

- When having a cold
- During exercise
- When laughing or weeping
- When playing or being outdoors
- In contact with furred animals
- Others________

21. In the past 12 months, how many attacks of wheezing have your child had?

- Never
- 1-3 times
- 4-12 times
- > 12 times

22. In the past 12 months, how often, on average, has your child’s sleep been disturbed due to wheezing

- Never woken with wheezing
- Less than one night per week
- One or more nights per week

23. In the past 12 months, has wheezing ever been severe enough to limit your speech to only one or two words at a time between breaths?

- Yes
- No

24. In the past 12 months, has your child had a dry cough at night for more than two weeks, apart from a cough associated with a cold or chest infection?

- Yes
- No

25. Has your child been taken to a doctor due to wheezing or dry cough problem?

- Yes
- No

26. Has your child been diagnosed with asthma by a doctor?

- Yes
- No

27. Has your child had croup?

- Yes
- No

28. Has your child been diagnosed tuberculosis?

- Yes
- No

29a. Has your child been diagnosed with pneumonia by a doctor?

- Yes,
- No, if NO, skip to question 30a

29b. If Yes, the 1^st^ diagnosed at age ____

29c. If Yes, it has occurred

- Only once
- 2-3 times
- 4 or more

Questions concerning rhinitis or eye irritations for the child

30a. Has your child ever had a problem with sneezing, or a runny, or a blocked nose when he / she did not have a cold or a flu?

- Yes
- No. If No, skip to question 34

30b. If Yes, at what age did the problems first occur?

- Prior to 1 year of age
- At 1 – 2 years of age
- At 3 – 4 years of age
- At 5-6 years of age
- Past 6 years of age

31a. In the past 12 months, has your child had a problem with sneezing, or a runny, or a blocked nose when he / she did not have a cold or the flu?

- Yes
- No, if no, skip to question 34

31b. In which of the past 12 months, did this nose problem occur? (please tick any which apply)

- January
- February
- March
- April
- May
- June
- July
- August
- September
- October
- November
- December

32. In the past 12 months, how much did this nose problem interfere with your child’s daily activities?

- Not at all
- A little
- A moderate amount
- A lot

33. In the past 12 months, has this nose problem been accompanied by *itchy-watery eyes*?

- Yes
- No

34. Has your child been diagnosed with hay fever or allergic rhinitis by a doctor?

- Yes
- No

35a. In the past 12 months, how many times has your child had a cold?

- None
- 1-2 times
- 3-5 times
- 6 – 10 times
- More than 10 times
- Not know

35b. Which season does your child usually have common cold?

- Spring
- Summer
- Autumn
- Winter

35c. How long does usually a cold last?

- Less than 2 weeks
- 2 – 4 weeks
- More than 4 weeks

36. Has your child ever had inflammations of the ears?

- No
- Yes, 1 – 2 times
- Yes, 3 – 5 times
- Yes, more than 5 times

Questions concerning eczema for the child

37 a) Has your child ever had an itchy rash, which was coming and going for at least 6 months?

- Yes
- No. if No, skip to 39

37 b) If Yes, at what age did the problem first occur?

- Prior to 1 year of age
- At 1-2 years of age
- At 3-4 years of age
- At 5-6 years of age
- Past 6 years of age

37 c) Has this itchy rash at any time affected any of the following places: the fold of the elbows, behind the knees, in front of the ankles, under the buttocks, or around the neck, ears or eyes?

- Yes
- No

38 a) Has your child had this itchy rash at any time in the last 12 months?

- Yes
- No, if no, skip to question 39

38 b) In the last 12 months, how often, on average, has your child been kept awake at night by this itchy rash?

- Never
- Less than one night per week
- One or more nights per week

39. Has your child been diagnosed with eczema by a doctor?

- Yes
- No

Questions concerning reactions to food

40 a) Has the child at any time had allergic irritations such as eczema, nettle-rash, diarrhoea, swollen lips or eyes caused by the listed foods below?

- Yes
- No
- Not known

40 b) If Yes, which kind of foods?

- Milk or dairy products
- Eggs
- Fish
- Peanuts
- Nuts, almond
- Vegetables, e.g. tomatoes, carrots
- Flour (wheat, barley, rye, oat)
- Soya, peas, beans
- Fruit
- Seafood, e.g. crab.
- Other

Questions concerning antibiotic treatment of the child

41a. Has the child been taking medicines with antibiotic, e.g. penicillin?

More than one alternative possible

- No, never
- Yes, when 0 – 12 months old
- Yes, when 12 – 24 months old
- Yes, after 24 months old

41b. If Yes, the antibiotic is used for______________

42. If the child was taking antibiotic when 0 – 12 months old, how many treatments did he / she receive?

- 1 treatment
- 2 treatments
- 3 or more treatments

Questions concerning chronic “modern” disease of the child

43. Has your child had any of the following disease/disorders diagnosed?

|  | Doctor diagnosed? | | Use of medicine | | When diagnosed? |
| --- | --- | --- | --- | --- | --- |
|  | Yes | No | Yes | No |
| Diabetes |  |  |  |  |  |
| MBD (Minimal Brain Dysfunction) |  |  |  |  |  |
| ADHD (Attention Deficit Hyperactivity Disorder) |  |  |  |  |  |
| Autism |  |  |  |  |  |
| Asperger |  |  |  |  |  |
| Tourette’s syndrome |  |  |  |  |  |
| Chryptorchidism, and hypospadias |  |  |  |  |  |

Questions concerning the health of the rest of the family

44 a) Do asthma or allergic problems exist in the family?

- Yes
- No. If No, skip to question 45

44 b) If Yes, which kind of problems and for whom?

Biological father

- Asthma
- Allergic nose or eyes problems
- Eczema

Biological mother

- Asthma
- Allergic nose or eyes problems
- Eczema

Siblings

- Asthma
- Allergic nose or eyes problems
- Eczema

45. How many times have you had colds in your household the past year?

Father

- None
- 1 – 2 times
- 3 – 4 times
- 5 or more times
- Not known / not applicable

Mother

- None
- 1 – 2 times
- 3 – 4 times
- 5 or more times
- Not known / not applicable

Sibling

- None
- 1 – 2 times
- 3 – 5 times
- 6-10 times
- More than 10 times
- Not known / not applicable

Questions concerning health problems for one of the parents

46. Question 47 shall only be answered by one of the parents. State below which one of the parents that will answer the question.

- Mother
- Father

47. During the last 3 months, have you had any (one or more) of the following symptoms ? (Answer every question even if you have not had any symptoms!)

Fatigue

- Yes, often (every week)
- Yes, sometimes
- No, never

If Yes, do you believe that it is due to your home environment?

- Yes
- No

Feeling heavy-headed

- Yes, often (every week)
- Yes, sometimes
- No, never

If Yes, do you believe that it is due to your home environment?

- Yes
- No

Headache

- Yes, often (every week)
- Yes, sometimes
- No, never

If Yes, do you believe that it is due to your home environment?

- Yes
- No

Nausea / dizziness

- Yes, often (every week)
- Yes, sometimes
- No, never

If Yes, do you believe that it is due to your home environment?

- Yes
- No

Difficulties concentrating

- Yes, often (every week)
- Yes, sometimes
- No, never

If Yes, do you believe that it is due to your home environment?

- Yes
- No

Itching, burning or irritation of the eyes

- Yes, often (every week)
- Yes, sometimes
- No, never

If Yes, do you believe that it is due to your home environment?

- Yes
- No

Irritating, stuffy or runny nose

- Yes, often (every week)
- Yes, sometimes
- No, never

If Yes, do you believe that it is due to your home environment?

- Yes
- No

Hoarse, dry throat

- Yes, often (every week)
- Yes, sometimes
- No, never

If Yes, do you believe that it is due to your home environment?

- Yes
- No

Cough

- Yes, often (every week)
- Yes, sometimes
- No, never

If Yes, do you believe that it is due to your home environment?

- Yes
- No

Dry or flushed facial skin

- Yes, often (every week)
- Yes, sometimes
- No, never

If Yes, do you believe that it is due to your home environment?

- Yes
- No

Scaling/ itching scalp or ears

- Yes, often (every week)
- Yes, sometimes
- No, never

If Yes, do you believe that it is due to your home environment?

- Yes
- No

Hands dry, itching, red skin

- Yes, often (every week)
- Yes, sometimes
- No, never

If Yes, do you believe that it is due to your home environment?

- Yes
- No

1. The residence of the child

Most of the questions in this chapter concern the child’s main residence, i.e. where the child lives the majority of the time. If child live with grandparents, please answer the residential condition of his/her grandparents. Also we would like to find out information about the residence where the child was born.

48. Has the child lived at the present residence the whole of his/her life?

- Yes
- No, lived here since_________

49a. Is the child living more than 10 days per month at another residence?

- Yes
- No

49b. If Yes, whom does child live with_________?

Questions concerning the surrounding of the present residence

50. Where is the residence situated?

- Inner city area
- Suburban
- Rural area (countryside)
- Other

51. If the residence near to high way or main road?

- Yes
- No

52. Is the residence near to a farm/property where cattle are kept?

(E.g. cows, pigs, horses)

- Yes
- No

Questions concerning the present residence

53. In which kind of house is the child living in at the moment?

- Flat roof Pingfang (bungalow)
- Sloping roof Pingfang
- Low-rise apartment (≤ 7 floors)
- High-rise apartment (> 7 floor)
- Villa or row house

54. Can you approximately estimate the size of your residence?

- Smaller than 40 m2
- 41-60 m2
- 61 – 75 m2
- 76 – 100 m2
- 101 – 150 m2
- Larger than 150 m2

55. Can you state, approximately, the age of the residence?

- Less than 10 year
- 10-20 years
- 20-30 years
- 30-40 years
- 40-50 years
- More than 50 years
- Not known

56. Are there insulation layers attached to the outer walls?

- Yes,
- No
- Do not know

57. Do you rent the current residence?

- Yes,
- No

Questions concerning the child’s room（*Child’s room means child have most activities here day and night*）

58. In which room does the child sleep most of his/her sleeping time?

(Choose only one alternative!)

- The child’s own room
- Sharing bed room with siblings (brothers and sisters)
- Sleeping with parents or grandparents
- Others_________

59. Is the child’s room directed?

- East
- West
- South
- North
- Other___

Questions concerning the construction and material of the present residence

60. State which kind of flooring material there is in the different rooms in the residence?

Mark in appropriate box with a cross for each room.

The child’s room

- Linoleum
- PVC floor
- Wood
- Laminated wood
- Stones/tiles
- Cement
- Carpets
- Other / Not known

The parents’ room

- Linoleum
- PVC floor
- Wood
- Laminated wood
- Stones/tiles
- Cement
- Carpets
- Other / Not known

The living room

- Linoleum
- PVC floor
- Wood
- Laminated wood
- Stones/tiles
- Cement
- Carpets
- Other / Not known

The kitchen

- Linoleum
- PVC floor
- Wood
- Laminated wood
- Stones/tiles
- Cement
- Carpets
- Other / Not known

61a. Which kind of surface layer is on the walls in the child’s room?

More than one alternative possible.

- Painting
- Latex paint
- Wall paper
- Stone/tiles
- Wood
- Textile / woven fabric
- Lime
- Other

61b. What kind of windows exists in the child’s room

- Wooden framed
- Aluminium framed
- PVC framed

61c. Glass in frames are:

- Single pane
- Double pane
- Double pane gas filled

62a. Which kind of surface layer is on the walls in the parents’ room?

More than one alternative possible.

- Painting
- Latex paint
- Wall paper
- Stone/tiles
- Wood
- Textile / woven fabric
- Lime
- Other

62b. What kind of windows exists in the parents’ room

- Wooden framed
- Aluminium framed
- PVC framed

62c. Glass in frames are:

- Single pane
- Double pane
- Double pane gas filled

Questions concerning heating and ventilation in the present residence

63a. Which type of heating is there in the residence?

- Electric (heating) radiator
- Hot water (heating) radiator
- Underfloor heating
- Warm air (central) heating
- Coal or wood stove
- Kang or firewall
- Other
- No heating

63b. During the winter, how comfortable is the temperature in your home?

- About right
- Too hot
- Too cold

64a. Which kind of cooling system is there in your residence?

- Air conditioning unit
- Electric fans
- By opening windows
- Other

64b. During summer, how comfortable is the temperature in your home?

- About right
- Too hot
- Too cold

65. Which kind of ventilation system is there in the residence (multiple choice)?

- Natural ventilation without fans
- Natural ventilation with fans in kitchen
- Natural ventilation with fans in bathroom
- Natural ventilation with fans in bedroom
- Mechanical ventilation system

66. What type of fuel do you have for cooking?

- Coal
- Biomass/wood
- Gas
- Electric
- Others______

Questions concerning renovations and extensions done to the present building

67a Have any major renovations or extensions been done to the building?

- Yes
- No, Go to question 68
- Not known, Go to question 68

67b If Yes, when was the house rebuilt/renovated?

More than one alternative is possible.

- 1-2 years ago
- 3-4 years ago
- 5-6 years ago
- 7-8 years ago
- 9-10 years ago

67c If Yes, was the action taken due to problems with damp and mould in the building?

- Yes
- No
- Not known

68. Has the flooring material been changed in any of the rooms, stated below, during the first year of the child or during the 6 months prior to the birth of the child?

The child’s room

- Yes
- No
- Not known

The parents’ room

- Yes
- No
- Not known

Other room/s

- Yes
- No
- Not known

69. Have any of the rooms, stated below, been repainted during the first year of the child or during the 6 months prior to the birth of the child?

The child’s room

- Yes
- No
- Not known

The parents’ room

- Yes
- No
- Not known

Other room/s

- Yes
- No
- Not known

Questions concerning dampness problems, if any, in the present residence

70. Have you noticed any visible mould on the floor, walls or ceiling in any of the rooms stated below?

The child’s room

- Yes
- No
- Not known

The parents’ room

- Yes
- No
- Not known

Other room/s

- Yes
- No
- Not known

The bathroom

- Yes
- No
- Not known

71. Have you noticed any visible damp stains on the floor, walls or ceiling in any of the rooms stated below?

The child’s room

- Yes
- No
- Not known

The parents’ room

- Yes
- No
- Not known

Other room/s

- Yes
- No
- Not known

The bathroom

- Yes
- No
- Not known

72. Do you suspect any humidity/mould problem in (inside) the floor, walls or ceiling, which are not visible on (from) the inside of the residence?

- Yes
- No
- Not known

73. Are there any floor covering that are detached or discoloured/blackened in any of the rooms stated below?

The child’s room

- Yes
- No
- Not known

The parents’ room

- Yes
- No
- Not known

Other room/s

- Yes
- No
- Not known

The bathroom

- Yes
- No
- Not known

74. Have there been any flooding or other kinds of water damages in the rooms stated below?

The child’s room

- Yes,
- No
- Not known

The parents’ room

- Yes,
- No
- Not known

The other room

- Yes,
- No
- Not known

The bathroom

- Yes,
- No
- Not known

75. In the winter, does condensation or moisture occur on the inside, at the bottom, of windows (windowpanes) in any of the rooms stated below?

The child’s room

- No, never
- Yes, less than 5 centimetres
- Yes, 5 – 25 centimetres
- Yes, more than 25 centimetres
- Not known

The parents’ room

- No, never
- Yes, less than 5 centimetres
- Yes, 5 – 25 centimetres
- Yes, more than 25 centimetres
- Not known

The living room

- No, never
- Yes, less than 5 centimetres
- Yes, 5 – 25 centimetres
- Yes, more than 25 centimetres
- Not known

Questions to the parents concerning odour in the present residence

76a. Who responded 76b on odour perception?

- Father
- Mother
- Other______

76b. Have you during the last 3 months been bothered by any (one or more) of the odours, stated below, in your residence?

Stuffy “bad” smell

- Yes, frequently (weekly)
- Yes, sometimes
- No, never

Unpleasant smell

- Yes, frequently (weekly)
- Yes, sometimes
- No, never

Pungent smell

- Yes, frequently (weekly)
- Yes, sometimes
- No, never

Mouldy smell

- Yes, frequently (weekly)
- Yes, sometimes
- No, never

Tobacco smoke

- Yes, frequently (weekly)
- Yes, sometimes
- No, never

Dry air

- Yes, frequently (weekly)
- Yes, sometimes
- No, never

Humid air

- Yes, frequently (weekly)
- Yes, sometimes
- No, never

Questions concerning the child’s residence at birth

Below, three questions are given concerning the residence in which the child spent his / her first time after the birth. If you did not move since the child was born, please skip these questions.

77. In the child’s birth residence, what kind of floor covering is used?

- Linoleum
- PVC floor
- Wood
- Laminated wood
- Stones/tiles
- Cement
- Carpets
- Other / Not known

78. In the child’s birth residence, did you notice

Visible mould or damp stains on the floor, walls or ceiling

- Yes, frequently (weekly)
- Yes, sometimes
- No, never

Floor covering that were detached or discoloured/blackened

- Yes, frequently (weekly)
- Yes, sometimes
- No, never

Flooding or other kind of water damages

- Yes, frequently (weekly)
- Yes, sometimes
- No, never

Condensation or moisture on the inside, at the bottom, of windows (windowpanes) in the winter period

- Yes, frequently (weekly)
- Yes, sometimes
- No, never

Suspected damp and mould problems in (inside) the floor, walls or ceiling, which are not visible on (from) the inside

- Yes, frequently (weekly)
- Yes, sometimes
- No, never

79. Did any (one or more) of the odours, stated below, occur in the child’s birth residence?

Stuffy “bad” smell

- Yes
- No

Unpleasant smell

- Yes
- No

Pungent smell

- Yes
- No

Mouldy smell

- Yes
- No

Tobacco smoke

- Yes
- No

Dry air

- Yes
- No

Humid air

- Yes
- No

Questions concerning furred animals

80a Do you have any furred animals / pets in your present residence?

- Yes
- No. If No, skip to question 81a

80b If Yes, what kind and how many?

- Cat___
- Dog___
- Rodent (rabbit, hamster, rats, guinea pig, etc.)____
- Birds___
- Aquarium fishes, reptiles, etc.____
- Other furred animals____

81a Were there any furred animals / pets in the residence during the child’s first years, i.e. in the child’s birth residence?

- Yes
- No. If No, skip to question 82

81b If Yes, what kind and how many?

- Cat____
- Dog____
- Rodent (rabbit, hamster, rats, guinea pig, etc.)____
- Birds____
- Aquarium fishes, reptiles, etc.____
- Other furred animals____

82. Have you got rid off any furred animals / pets due to allergic illnesses in the family?

- Yes
- No

83. Have you refrained from procuring any furred animals / pets due to allergic illnesses in the family?

- Yes
- No

84. In your child’s first year of life did he/she have regular (at least once a week) contact with farm animals (e.g. cattle, pigs, goats, sheep or poultry)?

- Yes
- No

85. Has this child’s mother had regular (at least once a week) contact with farm animals (e.g. cattle, pigs, goats, sheep or poultry) while being pregnant with this child?

- Yes
- No

Questions concerning cleaning routines, etc. in the residence

86a. How often do you clean the floor in the child’s room?

- Every day
- Approx. twice a week
- Once a week
- Every second week
- Once a month
- Less frequent

86b. Have your cleaning routines changed due to allergies in the family?

- Yes
- No
- Not known

86c. Which of these methods has been used to clean the floor in the child’s room? *Circle all that apply.*

- Broom
- Mop
- Vacuum
- Other______

86d. If you use wet mop, do you use detergent?

- Yes
- No

87. How often do you open window for ventilation?

- Every day
- Approx. twice a week
- Once a week
- Every second week
- Once a month
- Less frequent

88. How often do you sun-cure bed sheets?

- Often
- Sometimes
- Never

89. On average how often do you or your family members use the following products?

Humidifier

- Everyday
- A few times per week
- A few times per month
- A few times per year
- Less than a few times per year
- Never

Ionizer

- Everyday
- A few times per week
- A few times per month
- A few times per year
- Less than a few times per year
- Never

Ozone generator

- Everyday
- A few times per week
- A few times per month
- A few times per year
- Less than a few times per year
- Never

Air cleaner/purifier unit

- Everyday
- A few times per week
- A few times per month
- A few times per year
- Less than a few times per year
- Never

Fresher/room deodorizer

- Everyday
- A few times per week
- A few times per month
- A few times per year
- Less than a few times per year
- Never

Detergents used in kitchen

- Everyday
- A few times per week
- A few times per month
- A few times per year
- Less than a few times per year
- Never

Detergents used in bathroom or washing room (for laundry and/or toilet)

- Everyday
- A few times per week
- A few times per month
- A few times per year
- Less than a few times per year
- Never

Furniture polish

- Everyday
- A few times per week
- A few times per month
- A few times per year
- Less than a few times per year
- Never

Bug or insect spray

- Everyday
- A few times per week
- A few times per month
- A few times per year
- Less than a few times per year
- Never

Candles or incenses

- Everyday
- A few times per week
- A few times per month
- A few times per year
- Less than a few times per year
- Never

Questions concerning tobacco smoking

90a. Does anyone in your family smoke?

- No. If No, skip to question 91
- Yes, mother
- Yes, father
- Yes, siblings
- Yes, other person

90b. If Yes, where in the residence does/do the person/s smoke?

(More than one alternative possible)

- Outside on the balcony / patio
- Inside home

90c. If inside home, how many do people smoke cigarettes totally?

- Less than 10 per day
- 10-20 per day
- More than 20 per day
- Do not know

91. Did any of the parents smoke during the child’s first year of life?

Mother

- - Yes
  - No

Father

- - Yes
  - No

92. Did any of the parents smoke during the pregnancy?

Mother

- Yes
- No

Father

- Yes
- No

Questions concerning pillows and bedding of your child

|  | At present | During the child’s first year of life |
| --- | --- | --- |
| 93. What kind of pillow does or did your child use? (tick as many boxes as apply) | - Foam - Buckwheat pillow - Feather - Other______ | - Foam - Buckwheat pillow - Feather - Other______ |
| 94. What kind of bedding does or did your child use? (tick as many boxes as apply) | - Cotton - Cellucotton - Feather - Other______ | - Cotton - Cellucotton - Feather - Other______ |

1. Questions concerning food habits

In media it is often indicated that our modern lifestyle cause allergy. No-one has yet been able to explain what is actually meant with this conception (term). For this reason we would like to make an attempt to get an understanding of how our modern lifestyles are related to our eating habits.

| 95. In the past 12 months, how often, on average, did your child eat or drink the following? | |
| --- | --- |
| Meat（e.g. beef, lamb, chicken, pork） | - Never or occasionally - 1-2 times per week - 3-4 times per week - 5-6 times per week - Once per day or more |
| Seafood | - Never or occasionally - 1-2 times per week - 3-4 times per week - 5-6 times per week - Once per day or more |
| Fruit | - Never or occasionally - 1-2 times per week - 3-4 times per week - 5-6 times per week - Once per day or more |
| Vegetables | - Never or occasionally - 1-2 times per week - 3-4 times per week - 5-6 times per week - Once per day or more |
| Pulses and grains | - Never or occasionally - 1-2 times per week - 3-4 times per week - 5-6 times per week - Once per day or more |
| Rice and wheat（e.g. noodles, bread, steamed bun） | - Never or occasionally - 1-2 times per week - 3-4 times per week - 5-6 times per week - Once per day or more |
| Eggs | - Never or occasionally - 1-2 times per week - 3-4 times per week - 5-6 times per week - Once per day or more |
| Milk | - Never or occasionally - 1-2 times per week - 3-4 times per week - 5-6 times per week - Once per day or more |
| Fast food（e.g. KFC） | - Never or occasionally - 1-2 times per week - 3-4 times per week - 5-6 times per week - Once per day or more |

1. Questions regarding the use of TV sets and computers in the house

96. During a normal week, how many hours a day (24 hours) does your child watch television?

- Less than 1 hour
- 1-3 hours
- 3-5 hours
- 5 hours or more

97. Outside school hours, how many times a week does your child engage in outdoor physical activities?

- Never or occasionally
- Once or twice per week
- Three or more times a week

98. Who filled out the questionnaire?

- Father
- Mother
- Other______

99. This survey is completed at (year)____(month)____(day)____

Further comments:__________________________

To further investigate the influence of your indoor environment on child’s health, we would like to inspect your residence and do medical exam for your child.

Would you like to take the home inspection?

- Yes
- No

Would you like to take your child to the hospital for free medical examination?

- Yes
- No

If “yes”, please inform us your contact information:

Mobile phone: __________________

Home address: __________________

Home phone: ___________________

E-mail________________

*Thank you for your participants!*
