# Supplementary material for: Pet keeping in childhood and asthma and allergy among children in Tianjin area, China
Source: PLoS One. 2018 May 16;13(5):e0197274. doi: 10.1371/journal.pone.0197274 (PMC5955563; doi:10.1371/journal.pone.0197274)
Supplement: S1 Table — (DOCX) [file pone.0197274.s003.docx]

**S1 Table. Prevalence (%) of asthma and allergy among children with different avoidance behaviors**

|  | Categories of avoidance behaviors | | | | | | | | | | | |
| --- | --- | --- | --- | --- | --- | --- | --- | --- | --- | --- | --- | --- |
|  | Total | | | Rural | | | Suburban | | | Urban | | |
|  | A^a^ | B^b^ | C^c^ | A | B | C | A | B | C | A | B | C |
| Current wheeze | **7.2** | 4.7 | 4.5 | **2.9** | 2.8 | 2.4 | **5.0** | 3.6 | 4.2 | **9.1** | 6.9 | 5.1 |
| Current dry cough | **16.0** | 11.9 | 13.3 | 8.2 | 8.2 | **9.4** | 11.0 | **12.7** | 11.6 | **20.4** | 16.4 | 14.9 |
| Diagnosed asthma | **8.9** | 4.2 | 3.8 | **3.5** | 2.9 | 2.2 | **4.9** | 3.8 | 2.9 | **12.3** | 6.1 | 4.6 |
| Current rhinitis | **39.2** | 28.8 | 28.1 | **25.1** | 23.2 | 16.3 | **31.3** | 26.9 | 25.3 | **49.2** | 38.0 | 33.0 |
| Diagnosed rhinitis | **18.2** | 9.5 | 7.7 | 5.4 | **6.0** | 2.5 | **11.2** | 9.0 | 7.0 | **25.6** | 14.7 | 9.6 |
| Current eczema | **21.2** | 18.1 | 12.6 | **18.2** | 15.6 | 8.0 | 16.0 | **20.2** | 11.2 | **24.1** | 19.9 | 14.2 |
| Diagnosed eczema | **46.2** | 39.9 | 38.0 | 30.4 | **34.6** | 23.9 | **41.0** | 38.0 | 34.9 | **55.8** | 48.9 | 43.4 |

^a^A: No current pet, due to avoidance behavior.

^b^B: Having pets at current home.

^c^C: No current pet, but not due to avoidance behavior.
