# Supplementary material for: Pet keeping in childhood and asthma and allergy among children in Tianjin area, China
Source: PLoS One. 2018 May 16;13(5):e0197274. doi: 10.1371/journal.pone.0197274 (PMC5955563; doi:10.1371/journal.pone.0197274)
Supplement: S2 Table — (DOCX) [file pone.0197274.s004.docx]

**S2 Table. Adjusted odds ratio of the number of furry pets keeping and asthma and allergy among children^a^, stratified by locations**

|  |  | Current wheeze | Current dry cough | Diagnosed asthma | Current rhinitis | Diagnosed rhinitis | Current eczema | Diagnosed eczema |
| --- | --- | --- | --- | --- | --- | --- | --- | --- |
|  | No pet keeping (Reference) | 1 | 1 | 1 | 1 | 1 | 1 | 1 |
| Currently |  | Rural | | | | | | |
|  | One furry pet^b^ | ----- | 0.48(0.16-1.41) | ----- | **2.31(1.13-4.71)** | **9.44(1.91-46.53)** | **6.95(2.68-18.05)** | **2.50(1.27-4.90)** |
|  | Two or more furry pets | ----- | 0.47(0.15-1.51) | ----- | 1.52(0.69-3.36) | **8.66(1.63-46.11)** | **4.37(1.55-12.32)** | **3.09(1.52-6.28)** |
|  |  | Suburban | | | | | | |
|  | One furry pet | 0.64(0.12-3.34) | 0.98(0.37-2.61) | 2.21(0.47-10.40) | 1.27(0.62-2.58) | 2.16(0.72-6.46) | 1.82(0.72-4.62) | 0.96(0.49-1.89) |
|  | Two or more furry pets | 2.02(0.38-10.72) | 1.01(0.30-3.40) | 1.15(0.11-12.30) | 0.40(0.12-1.31) | 2.85(0.72-11.27) | **3.24(1.10-9.53)** | 0.94(0.40-2.25) |
|  |  | Urban | | | | | | |
|  | One furry pet | 2.06(0.86-4.90) | 1.67(0.96-2.90) | **5.21(2.32-11.73)** | **1.78(1.13-2.81)** | **3.92(2.08-7.38)** | **1.86(1.04-3.31)** | **1.75(1.13-2.71)** |
|  | Two or more furry pets | 3.03(0.91-10.15) | 2.03(0.89-4.59) | **4.74(1.41-15.97)** | **2.12(1.05-4.31)** | 2.66(0.92-7.70) | **2.89(1.24-6.72)** | 1.80(0.91-3.59) |

S2 Table (Conntinued)

|  |  | Current wheeze | Current dry cough | Diagnosed asthma | Current rhinitis | Diagnosed rhinitis | Current eczema | Diagnosed eczema |
| --- | --- | --- | --- | --- | --- | --- | --- | --- |
|  | No pet keeping (Reference) | 1 | 1 | 1 | 1 | 1 | 1 | 1 |
| Early |  | Rural | | | | | | |
|  | One furry pet | 1.63(0.57-4.71) | 0.90(0.50-1.62) | 1.80(0.75-4.32) | 1.09(0.73-1.64) | 1.78(0.82-3.85) | **2.14(1.31-3.51)** | 1.39(0.97-2.00) |
|  | Two or more furry pets | ------ | 1.16(0.02-1.24) | 1.11(0.23-5.31) | 0.73(0.36-1.51) | 1.48(0.41-5.40) | 2.14(0.96-4.77) | 1.19(0.65-2.17) |
|  |  | Suburban | | | | | | |
|  | One furry pet | 1.94(0.73-20.36) | 0.90(0.46-1.78) | 0.51(0.11-2.29) | 0.90(0.54-1.51) | 1.65(0.76-3.58) | 0.93(0.48-1.81) | 0.56(0.34-0.92) |
|  | Two or more furry pets | 3.99(0.79-20.36) | 1.65(0.52-5.20) | 3.90(0.78-19.41) | 0.16(0.02-1.21) | **5.19(1.59-16.94)** | 0.73(0.16-3.26) | 0.64(0.24-1.70) |
|  |  | Urban | | | | | | |
|  | One furry pet | **2.13(1.18-3.85)** | **1.77(1.19-2.65)** | 1.25(0.62-2.51) | 1.25(0.87-1.78) | 1.33(0.81-2.20) | 1.04(0.66-1.63) | 0.99(0.71-1.39) |
|  | Two or more furry pets | 2.57(0.95-6.95) | **2.15(1.06-4.36)** | 0.95(0.22-4.10) | 0.62(0.30-1.27) | 0.56(0.17-1.90) | 0.85(0.35-2.08) | 1.09(0.58-2.06) |

^a^No pet keeping was set as reference. Odds ratio was adjusted for gender, age, total income, family allergic history, home dampness and avoidance behavior.

^b^Furry pet: cats, dogs, rodents and birds.
